# Injection of human umbilical cord mesenchymal stem cells exosomes for the treatment of knee osteoarthritis: from preclinical to clinical research

**DOI:** 10.1186/s12967-025-06623-y

**Published:** 2025-06-11

**Authors:** Yuzhong Wang, Yajie Kong, Jiejie Du, Lifei Qi, Meiling Liu, Siyi Xie, Jianghui Hao, Ming Li, Shuxing Cao, Huixian Cui, Aijing Liu, Jun Ma, Yongzhou Song

**Affiliations:** 1 Present Address: Department of Orthopedics, The Second Hospital of Hebei Medical University, Shijiazhuang, Hebei Province 050000 P. R. China; 2 Hebei Key Laboratory of Rare Disease, Shijiazhuang, Hebei Province 050000 P. R. China; 3 Hebei Medical University-University of Galway Stem Cell Research Center, Hebei Medical University, Shijiazhuang, Hebei Province 050017 P. R. China; 4 Department of Neurology, The Second Hospital of Hebei Medical University, Shijiazhuang, Hebei Province 050000 P. R. China; 5 Hebei Taihe Yunshan Biotechnology Co., Ltd., Shijiazhuang, Hebei Province P. R. China; 6 Department of Rheumatology and Immunology, The Second Hospital of Hebei Medical University, Shijiazhuang, Hebei Province 050000 China; 7 Hebei Technology Innovation Center for Stem Cell and Regenerative Medicine, Shijiazhuang, Hebei Province 050017 China; 8 Hebei Research Center for Stem Cell Medical Translational Engineering, Hebei Province 050017 Shijiazhuang, China

**Keywords:** Osteoarthritis, Exosomes, Human umbilical cord mesenchymal stem cells, Clinical research

## Abstract

**Background:**

Background: Exosomes (Exos) derived from mesenchymal stem cells (MSCs) share similar biological functions with MSCs but are more stable under various pathophysiological conditions, with a lower risk of immune rejection. Human umbilical cord mesenchymal stem cells (hUC-MSCs) are a promising source of MSC-derived exosomes (MSC-Exos), particularly for the treatment of osteoarthritis (OA), a degenerative joint disease characterized by inflammation and cartilage damage.

**Methods:**

In this study, we conducted in vitro experiments on mouse articular chondrocytes and treated mouse OA models with hUC-MSCs-Exos. To validate the results of hUC-MSCs-Exos in humans, a randomized, double-blind, ascending dose study was conducted to investigate the safety and efficacy of hUC-MSCs-Exos in the treatment of OA, and human chondrocyte toxicity experiments were conducted prior to the clinical trial.

**Results:**

In this study, we successfully extracted hUC-MSCs and verified their multilineage differentiation ability in different culture media. We then verified the Exos morphology and the expression of CD9, CD63, TSG1, and CALN. In preclinical experiments in vitro and in vivo, we verified that hUC-MSC- Exos can reduce the inflammatory response of articular cartilage and promote its regeneration. Finally, clinical experiments confirmed that hUC-MSC- Exos injection treatment of OA patients did not cause any adverse consequences, and a certain degree of effectiveness was found in the comparison of clinical scores and MRI examinations before and after treatment.

**Conclusions:**

This study shows that hUC-MSC-derived exosomes effectively reduce inflammation and promote cartilage regeneration in osteoarthritis, with demonstrated safety and efficacy in both preclinical and clinical settings.

**Trial registration:**

Trial No.MR-13-24-017929. Registered 11 February 2023, https://medicalresearch.org.cn/login#.

**Supplementary Information:**

The online version contains supplementary material available at 10.1186/s12967-025-06623-y.

## Introduction

Osteoarthritis (OA) is a chronic degenerative joint disease that affects the entire joint tissue, including articular cartilage, synovium, and subchondral bone [1]. Data from the Global Burden of Disease Study in 2017 show that musculoskeletal disorders are the leading cause of disability in middle-aged and elderly individuals. Among these, OA is the second most important cause of disability, following neck and back pain. In the United States, approximately 27 million people are affected by this disease, and related medical costs are estimated to exceed $185 billion annually [2, 3]. Recent studies indicate that inflammation and the catabolic processes it triggers are the primary causes of joint tissue damage, exacerbation of arthritis symptoms, and acceleration of degeneration [4].

Due to the complex pathogenesis of OA, there are currently few effective treatment strategies to improve joint balance, delay the progression of osteoarthritis, and promote cartilage repair [5, 6]. In clinical practice, the current conservative treatment methods have failed to repair joint tissue damage, while surgical treatments are associated with physical trauma and numerous unpredictable risks [7, 8]. Therefore, there is an urgent need to develop new therapeutic approaches to meet future clinical treatment demands.

In recent years, exosome-based regenerative therapies have gained widespread attention in both basic and clinical research [9]. An increasing number of studies indicate that exosomes derived from stem cells have the same effect as stem cell transplantation in slowing the progression of OA [10]. Exosomes are nano-sized membrane-bound carriers, ranging from 50 to 150 nanometers in diameter, with immunologically inert properties, providing a new option for the treatment of OA [11]. Previous studies have isolated and extracted exosomes from various human tissues (such as bone marrow, umbilical cord, and adipose tissue), confirming their potent anti-inflammatory, anti-fibrotic, and pro-angiogenic remodeling capabilities [12].

Its main mechanism of action is through the P38, ERK, AKT, HDAC3, and NF-KB pathways, with exosome-carried microRNAs involved in the anti-inflammatory process [13–17]. Several studies suggest that the inflammation induced by interleukin-6 (IL-6) leads to the detachment of articular cartilage through multiple mechanisms, with matrix metalloproteinase (MMP) 13 expression significantly increased in osteoarthritis tissues [18]. As the levels of inflammatory factors rise, the chondrogenic marker type II collagen (COL2A1) in chondrocytes significantly decreases, indicating that the secretion of inflammatory factors such as IL-6 and MMP 13 accelerates chondrocyte detachment and inhibits regeneration [19, 20]. Human umbilical cord mesenchymal stem cells (hUC-MSCs), which are extracted from discarded umbilical cord tissue, have the advantage of being easily accessible and are considered an important source of MSC-derived exosomes (MSCs-Exos) [21]. Recent research evidence supports the therapeutic potential of hUC-MSCs in arthritis diseases, which is related to the reduction of inflammatory molecules and the increase in anti-inflammatory molecules. hUC-MSCs can rapidly mobilize surface chondrocytes to the damaged areas, initiate the repair process, delay cell apoptosis, and promote cartilage regeneration [22]. hUC-MSC-exosomes (hUC-MSC-Exos) exhibit similar biological activity to hUC-MSCs, and several studies have demonstrated that hUC-MSC-Exos can slow the progression of OA through at least three mechanisms: first, by reducing pro-inflammatory factor secretion and decreasing osteosclerosis; second, by increasing the expression of COL2A1 and cluster gel; and third, by inhibiting the overexpression of ADAMTS5 and MMP13 [18, 19].

**Fig. 1:**
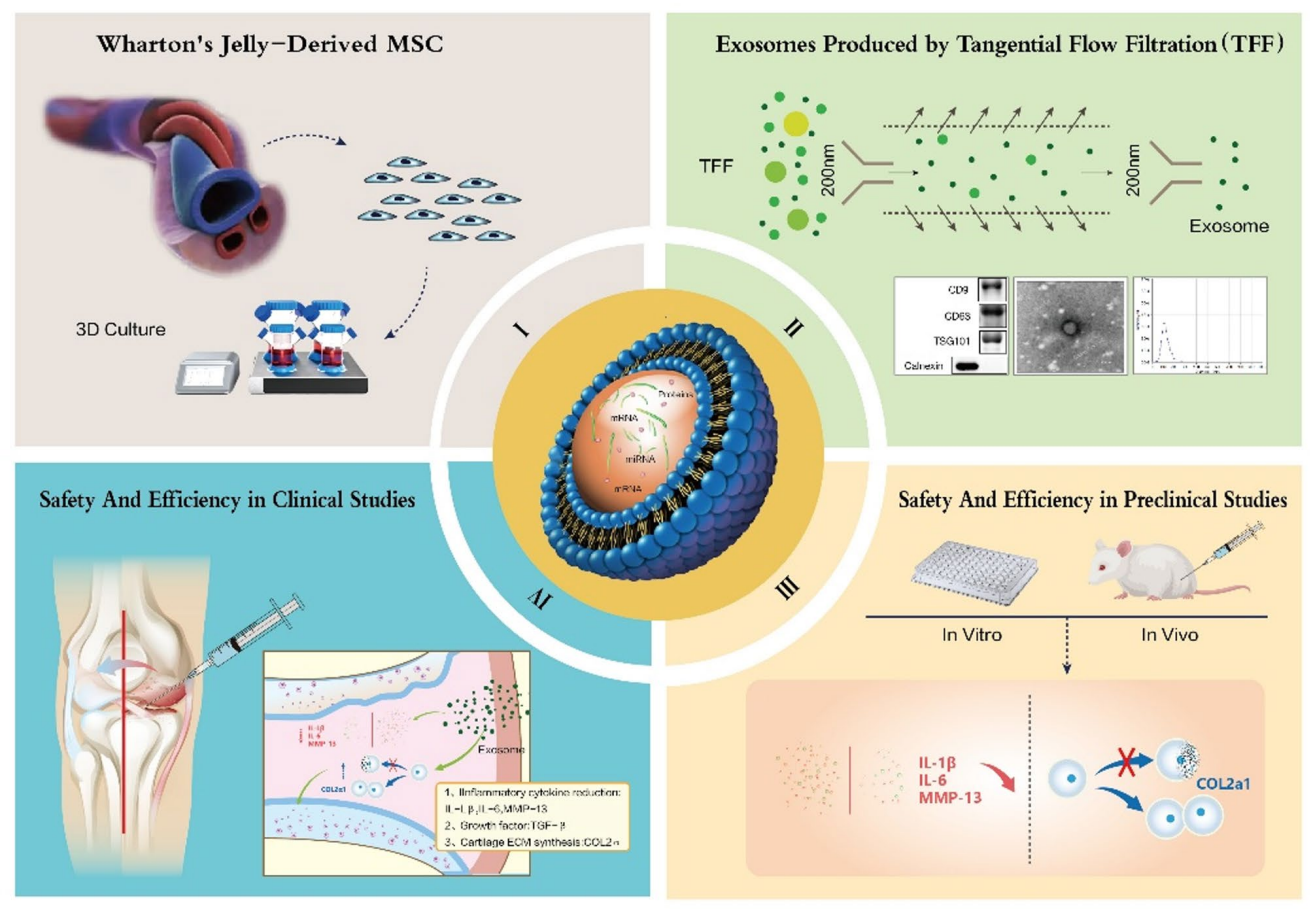
In this study, we aim to explore the safety and efficacy of hUC-MSC-Exos in the treatment of osteoarthritis through three parts: cell experiments, animal experiments, and clinical trials

In this study, we conducted in vitro experiments on mouse chondrocytes and treated a mouse OA model with hUC-MSCs-Exos. Subsequently, to validate the results of hUC-MSCs-Exos in humans, we validated their cytotoxicity in human chondrocytes and conducted a randomized, double-blind, escalating dose clinical study, aimed at exploring the safety and efficacy of hUC-MSCs-Exos in the treatment of OA (See Fig. 1).

## Results and discussion

### Isolation and identification of hUC-MSC-Exos

#### Extraction of human umbilical cord blood samples

**Fig. 2:**
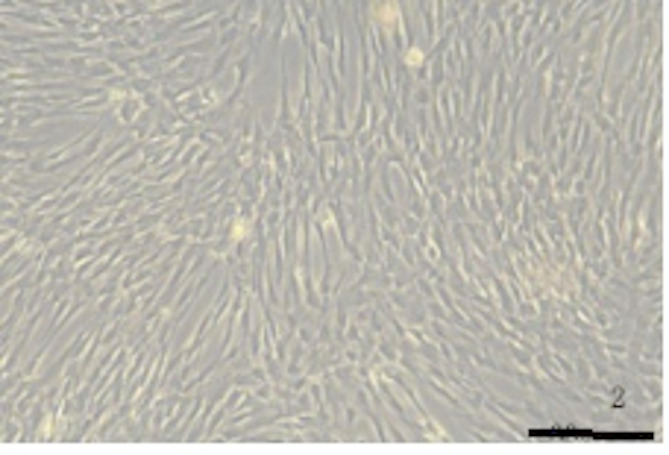
Morphological characteristics of hUC-MSC: The cells are spindle-shaped, fibroblast-like, and have clear cell edges

In the study by Julianna Kobolak et al. on the characterization and biological properties of MSCs, the morphology of MSCs is similar to that of fibroblasts [23]. Specifically, umbilical cord mesenchymal stem cells have a spindle-shaped (fibroblast-like) morphology, with abundant cytoplasm and large nuclei [24]. We observed that MSCs derived from umbilical cord tissue exhibited a spindle, fibroblast-like morphology, with clear cell boundaries (see Fig. 2), which is consistent with the literature reports.

#### Cell isolation and identification

**Fig. 3:**
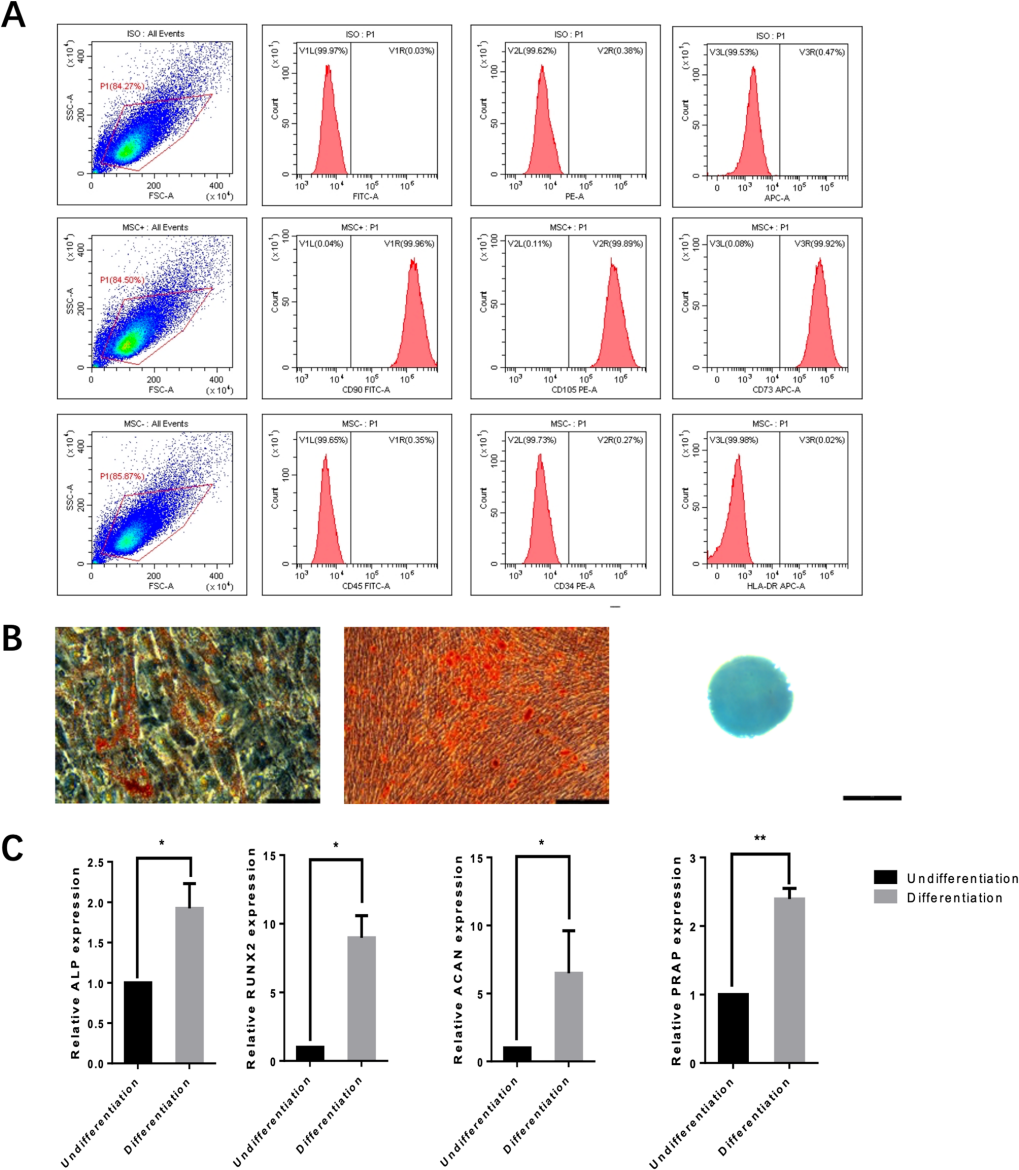
(**a**) Flow cytometry analysis of phenotypic marker expression; (**b**) Alizarin red staining (200x): Observation of mineralized nodules after 3 weeks of osteogenic induction (stained red); Safranin O staining (400x): Observation of lipid droplets formed after 3 weeks of adipogenesis (stained orange-red); Alisin blue staining (40x): Observation of chondrogenic spheres formed after 3 weeks of chondrogenic induction (stained blue). (**c**) ALP, Runx2, ACAN, and PPARγ were all well expressed through differentiation induction

It has been reported that hUC-MSCs are positive for CD13, CD29, CD73, CD90, CD105, and HLA-ABC, and negative for CD34, CD45, CD133, and HLA-DR [25, 26]. In our experiment, flow cytometry analysis showed that hUC-MSCs were positive for CD73, CD90, and CD105 (> 98%), and negative for CD45, CD34, and HLA-DR (≤ 2%) (see Fig. 3a). Osteogenic potential was evaluated using alkaline phosphatase (ALP) staining, adipogenic potential was assessed with Oil Red O staining, and chondrogenic potential was evaluated by Safranin O-fast green staining (see Fig. 3b). The osteogenic regulators were ALP and Runx2, while ACAN regulates chondrogenesis [27–29], and PPARγ regulates adipogenesis. Subsequently, RT-PCR was performed to assess the multi-lineage differentiation potential of MSCs. The results showed that mineralized nodules after 3 weeks of osteogenic induction could be observed with Alizarin Red staining (red); lipid droplets formed after 3 weeks of adipogenic induction were observed with Oil Red O staining (orange-red); and chondrogenic pellets formed after 3 weeks of chondrogenic induction were observed with Alcian Blue staining (blue). ALP, Runx2, ACAN, and PPARγ were well expressed following differentiation induction (see Fig. 3c). These data indicate that hUC-MSCs were successfully isolated in vitro and have the ability to differentiate into bone, cartilage, and adipose tissue.

#### Exosome extraction and identification

**Fig. 4:**
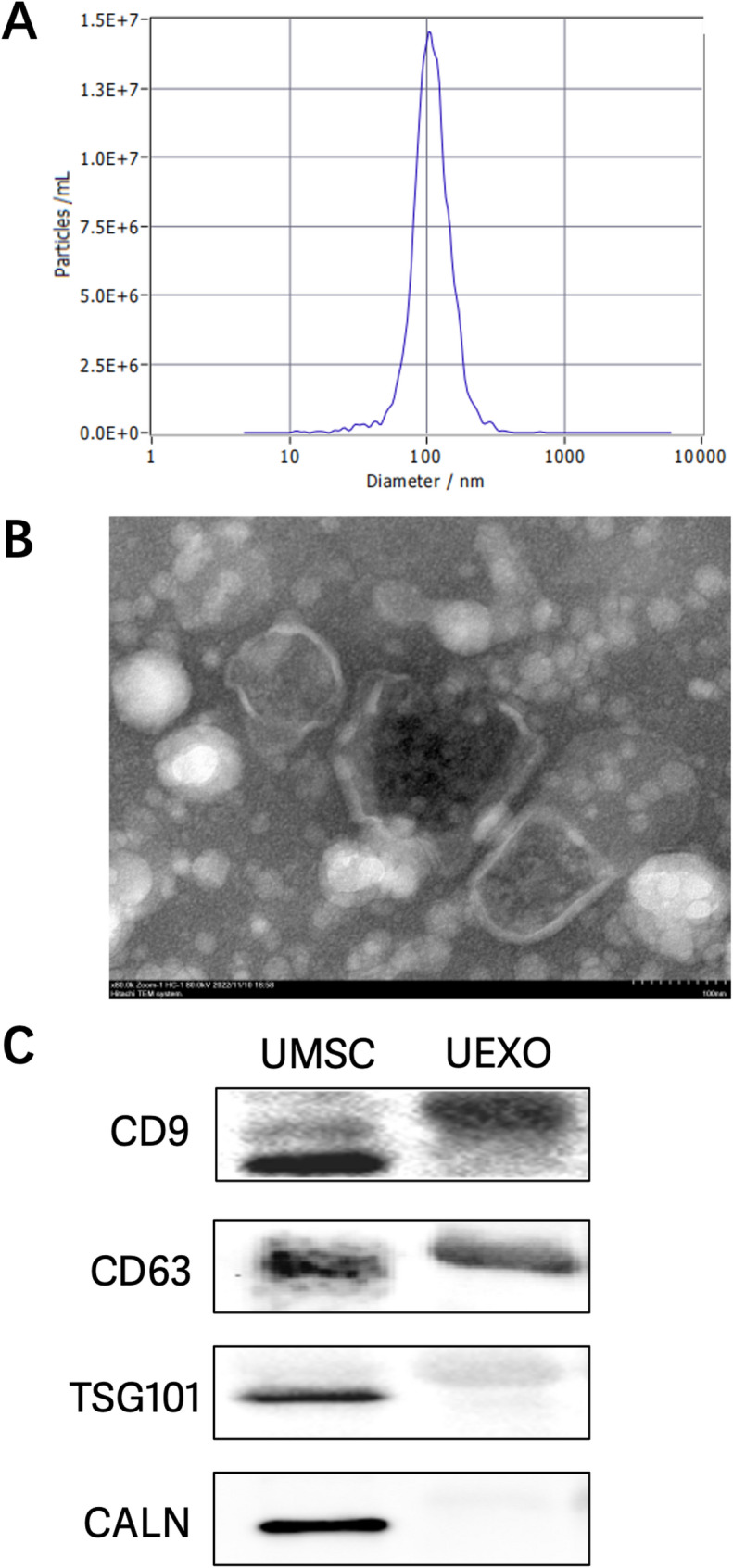
(**a**) The concentration and particle size of Exos were analyzed by NTA. (**b**) Exos were observed to be cup-shaped or spherical by transmission electron microscopy. (**c**) Surface exosome markers (CD63, TSG101, and CD9) of Exos were detected by immunoblotting

**Exosome**(Exos) are nano-sized (50–200 nm) extracellular vesicles produced via the endocytic pathway [30], and are secreted by many cells in response to their surrounding environment. CD9 and CD63 are important Exos markers, highly enriched in Exos [31], while Tumor Susceptibility Gene 101 (TSG101) plays a key role in the biogenesis and secretion of Exos [32]. Additionally, leptin enhances Exos release by increasing TSG101 expression [33]. During the process of Exos isolation, we found that hUC-MSC-Exos had a diameter of approximately 100 nm, consistent with the typical size range for Exos (Fig. 4a), following the guidelines recommended by ISEV (International Society for Extracellular Vesicles), known as MISEV2018 (Minimal Information for Studies of Extracellular Vesicles 2018) [34]. Negative-staining transmission electron microscopy (TEM) showed that the Exos had a cup-like or spherical shape, with a lipid bilayer membrane, giving them a distinct vesicular structure (Fig. 4b), similar to the morphology reported in previous literature [35]. Furthermore, Western blot analysis showed that Exos were positive for CD9, CD63, and TSG101 expression, while CALN was negative (Fig. 4c), confirming that we successfully isolated Exos from hUC-MSCs.

### Application of hUC-MSC-exos in the treatment of OA

hUC-MSCs are sourced from umbilical cord tissue, with a non-invasive cell collection process, relatively few ethical conflicts, low risk of teratoma formation, and the ability to differentiate into multiple lineages [36]. They are considered an ideal source of stem cells. A large body of research has confirmed the immunoregulatory potential of MSCs in inflammatory diseases such as OA [37–39]. MSC-Exos, as a paracrine secretion product, represents a safer and more effective means of regulating joint cavity inflammation and immunity in OA patients. Increasing evidence suggests that the significant therapeutic contribution of MSCs is the result of their paracrine effects [40, 41], and Exos are key paracrine regulatory factors involved in MSC-mediated tissue repair [42]. Recently, more studies have explored the anti-inflammatory potential of MSC-Exos. It has been reported that MSC-Exos derived from bone marrow and synovium can suppress inflammation in chondrocytes [43] and exert anti-inflammatory effects by modulating the biological behavior of macrophages. Shen et al. reported that the highly expressed C-C chemokine receptor-2 in Exos can bind to and inhibit the activity of the pro-inflammatory chemokine ligand 2 (CCL2), thereby effectively preventing macrophage accumulation and reducing the inflammatory response [44]. These findings inspire us, as Exos share similar biological effects with MSCs.

### The effect of hUC-MSC-Exos on mouse chondrocytes

IL-1β is one of the key factors that induce inflammation and extracellular matrix (ECM) degradation in articular cartilage [45], specifically by inhibiting the expression of ECM synthesis proteins (such as aggrecan and type II collagen) [45] and promoting the expression of matrix degrading proteins (MMP13 and ADAMTS5) [46]. Two major cytokines involved in the pathogenesis of OA, interleukin-1β (IL-1β) and tumor necrosis factor α (TNFα), are primarily produced by M1 synovial macrophage polarization, and they seem to drive inflammatory and destructive responses in synovial fibroblasts [47, 48]. These two cytokines synergistically activate other major pro-inflammatory mediators (IL-6, IL-10, prostaglandin F2) and proteolytic enzymes (MMP1, MMP3, MMP13, and integrins and metalloproteinases such as ADAMTS4 and ADAMTS5) in the joint [49, 50].

**Fig. 5:**
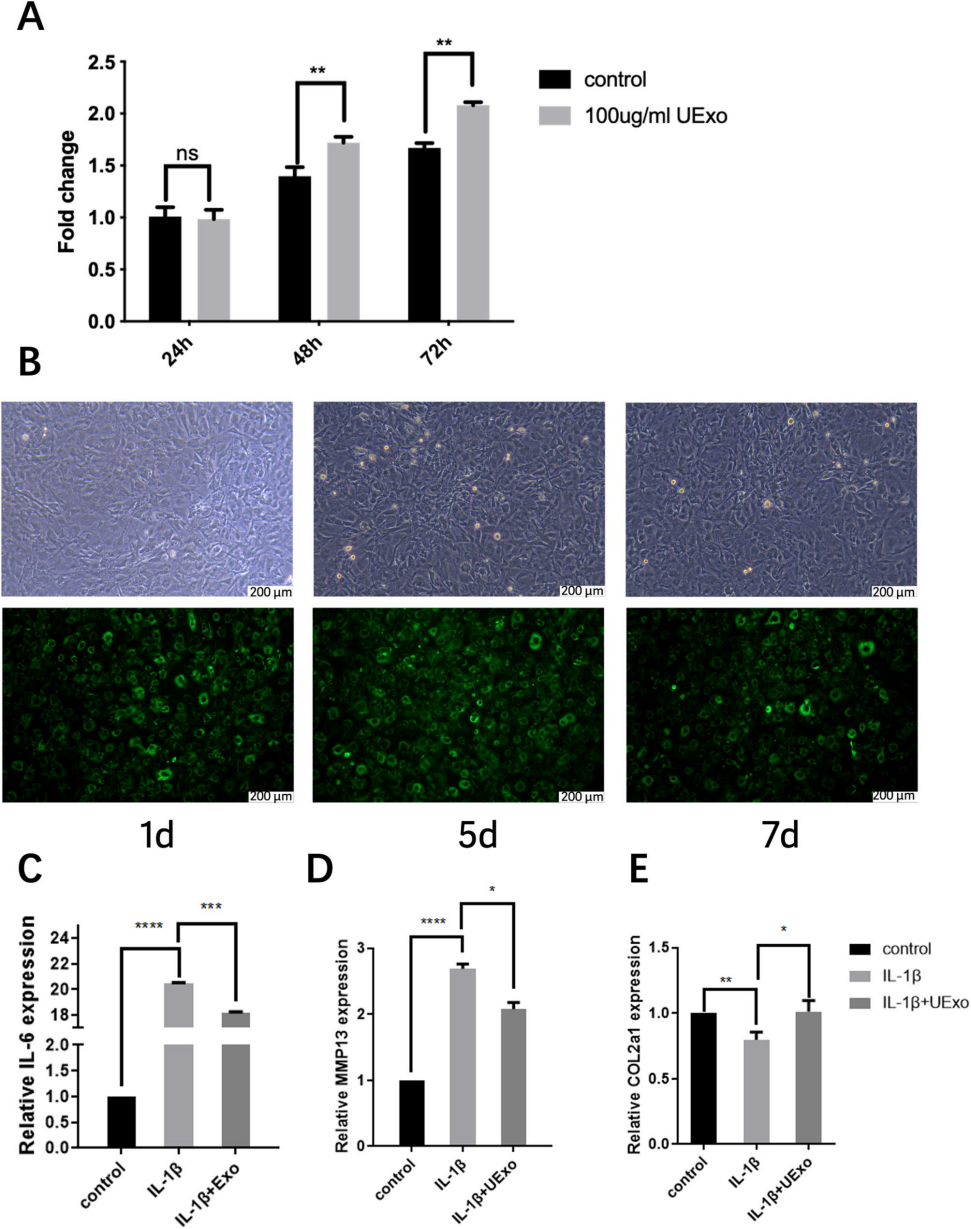
The effect of hUC-MSC-derived Exos on chondrocytes. (**a**) Compared to the control group, chondrocyte proliferation was observed at 24, 48, and 72 h after UExo treatment. (**b**) Representative images under fluorescence microscopy show UExos internalized by chondrocytes (100x magnification). IL1β (10 ng/ml) was used to induce normal chondrocytes for 24 h, promoting mRNA expression of IL-6 (**c**) and MMP13 (**d**), while inhibiting mRNA expression of Col2α1 (**e**). At the same time, treatment with hUC-MSC-derived Exos significantly inhibited the mRNA expression of IL-6 (**c**) and MMP13 (**d**), and promoted the mRNA expression of Col2α1 (**e**). * *P* < 0.05, ** *P* < 0.01 (mean ± standard deviation, *n* = 3)

MSC can effectively regulate macrophage polarization in the body [51]. Recent evidence suggests that hUC-MSCs-Exos also have the ability to reduce inflammation and promote osteochondral regeneration [52]. MSC-Exos secrete various immune-regulatory and repair molecules. MSC-Exos can redirect the polarization of macrophages in the joint to a more immunoregulatory state, thereby reducing inflammation and aiding in chondrocyte repair [53, 54]. In our experiment, cell viability was significantly higher in the hUC-MSC-Exos treatment group compared to the control group, and the cell viability in the treatment group did not significantly decrease over time. Therefore, we found reason to believe that hUC-MSC-Exos may promote chondrocyte proliferation (Fig. 5a). When Dio-labeled Exos were injected into chondrocytes, green fluorescence expression was detected at different intervals throughout the experiment and persisted for a longer period. This suggests that hUC-MSC-Exos can persist in cartilage tissue (Fig. 5b).

In inflammation assessment, the IL-6 cytokine is associated with both pro-inflammatory and anti-inflammatory actions. Previous studies have shown that the IL-6/IL-8 ratio plays a crucial role in the specific polarization of the cellular microenvironment [55]. Most MMPs, including MMP13, are involved in the renewal of osteoarticular epithelium, with MMP13 playing a dominant role in the process of cartilage destruction [56]. Additionally, ferroptosis occurs in chondrocytes in inflammation and iron overload. Induction of ferroptosis leads to increased expression of MMP13 and decreased expression of COL2A1 in chondrocytes [57]. In our IL1β-induced cartilage cell injury model, treatment with hUC-MSC-Exos reduced the expression of inflammatory factors (IL6, Fig. 5c) and MMP13(Fig. 5d) in chondrocytes. Furthermore, hUC-MSC-Exos treatment effectively increased the expression of COL2A1(Fig. 4e). Our findings also confirm that hUC-MSCs-Exos, on the one hand, can counteract IL-1β-induced chondrocyte inflammation, and on the other hand, can increase COL2A1 expression and reduce IL-6 and MMP13 levels, completing the anti-inflammatory effects on chondrocytes. In our experiment, we observed that hUC-MSC-Exos can be internalized by chondrocytes and inhibit the expression of some inflammatory factors and matrix degradation proteins. This may be related to the reduction of macrophage depolarization within the joint, leading us to believe that hUC-MSC-Exos is an effective means to reduce cellular inflammation and promote cartilage regeneration. However, further studies are needed to explore the underlying mechanisms.

### The effect of hUC-MSC-Exos on cartilage repair in OA mouse models

Based on the cell experiments, we conducted in vivo animal studies following the planned methodology (see later sections). OA mouse models were induced by intra-articular injection of chemical agents [58]. At the 5th week after modeling, hUC-MSC-Exos were injected into the joint cavities of the experimental group mice. At the 8th week after OA induction, knee joint specimens were collected for further analysis. It was observed that the overall appearance showed that, compared to the normal group, the control group had significantly rougher joint surfaces with local erosion, indicating the successful establishment of the OA model. Additionally, we observed that the cartilage in the hUC-MSC-Exos group was generally repaired to some extent. Compared to the control group, the joint surface in the hUC-MSC-Exos group was smoother.

**Fig. 6:**
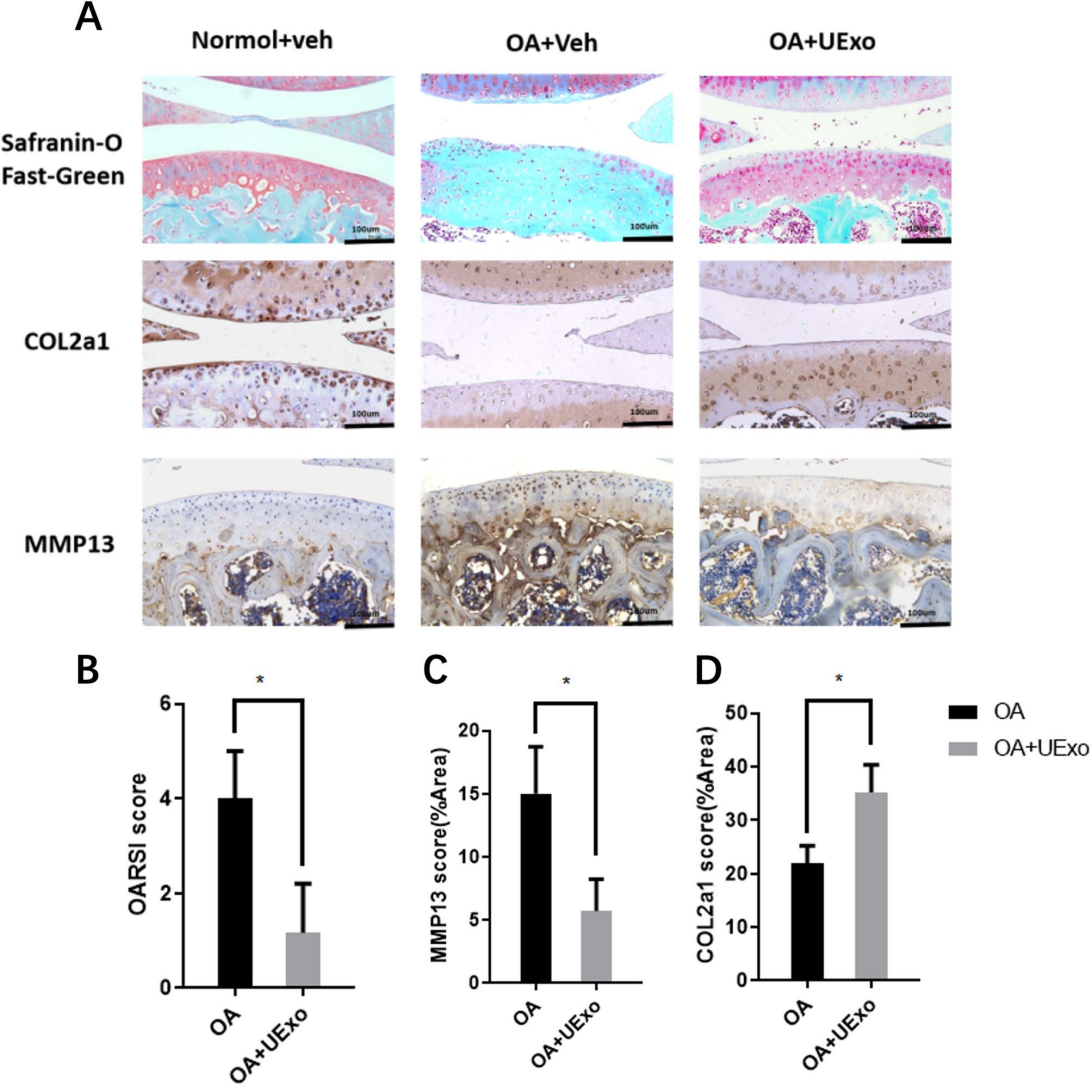
The effect of hUC-MSC-Exos on osteoarthritis mice. (**a**) H&E staining, Safranin O-fast green staining, and immunohistochemistry (IHC) staining images (200x). (**b**) Statistical analysis of OARSI scores for the OA group and OA group + UExo group (*p* ≤ 0.05). ImageJ software was used to quantitatively compare the IHC results: (**c**) Expression of MMP13(*p* ≤ 0.05); (**d**) Expression of COL2A1 in different groups (*p* ≤ 0.05)

During the pathogenesis of OA, IL-1β induces the expression of MMPs, which leads to severe damage of the ECM, including the loss of proteoglycans and COL2A1 [59]. We evaluated the OARSI (Osteoarthritis Research Society International) grading system [60] using Safranin O-fast green staining images (Fig. 6a). The results showed that the normal group had smooth cartilage surfaces, clear structure, and uniform positive staining. The control group exhibited the most severe cartilage damage, including cartilage erosion, subchondral bone thickening, abnormal distribution of chondrocytes, and loss of proteoglycans. The OARSI score of the hUC-MSC-Exos group was significantly lower than that of the OA group (Fig. 6b; Table 1). In contrast, when hUC-MSC-Exos were injected, the cartilage showed good reconstruction, characterized by a regular surface, restored cartilage thickness, and chondrocyte morphology close to normal. According to the OARSI grading, the OA group had a score of 6, while the hUC-MSC-Exos treatment group had a score of 1, indicating a significant therapeutic effect (Fig. 6b; Table 1). This suggests that the Exos effectively reduced cartilage damage during OA progression in rats and even promoted chondrocyte regeneration.

**Table 1:** Quantitative analysis results of OA and OA+UExo

Quantitative indicators	OA	OA+UExo
OARSI	3\4\5	1.5\2.0\0
MMP13	11.398\19.853\14.779	4.618\8.6\3.994
COL2A1	18.48\25.154\22.038	30.053\40.302\35.304

To further explore the effect of exosome treatment on the in vivo cartilage matrix, we investigated the potential mechanisms by which hUC-MSC-Exos alleviate OA. Immunohistochemical staining was used to assess the expression of COL2 and MMP13 [61, 62]. The results showed that, compared to the control group, the hUC-MSC-Exos group exhibited increased COL2A1 expression and decreased MMP13 expression (Fig. 6; Table 1). These data suggest that the mouse model is widely used in in vivo studies of cartilage degradation, primarily exhibiting pathological changes such as joint space narrowing, cartilage erosion, and surface calcification. Histological staining indicated that hUC-MSC-Exos treatment could prevent cartilage degradation and reduce the OARSI score in mice, reversing the progression of OA. The in vivo results were consistent with the in vitro findings, suggesting that hUC-MSC-Exos may regulate cartilage homeostasis by controlling the synthesis and degradation of the matrix in chondrocytes.

### The effect of hUC-MSC-Exos on human chondrocytes

**Fig. 7:**
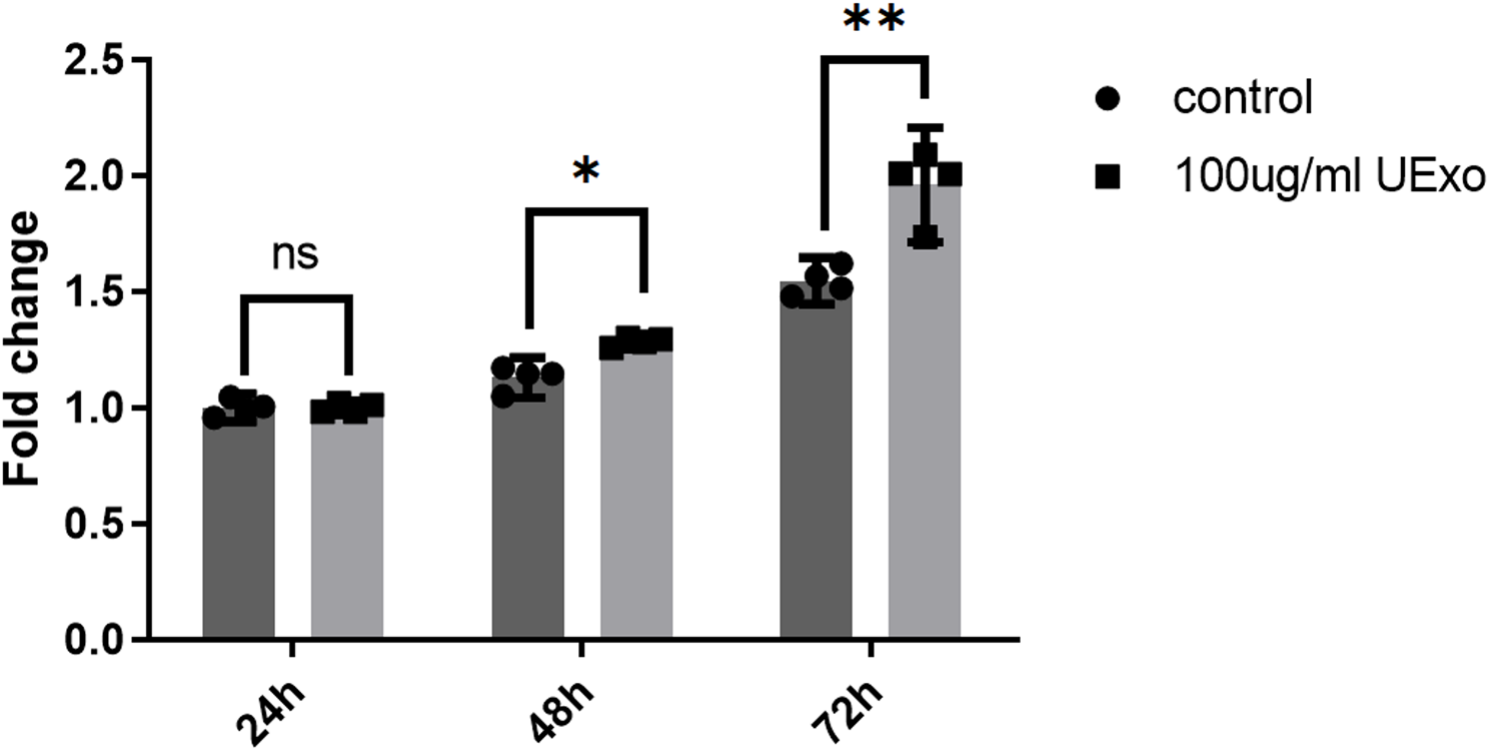
Effect of hUC-MSC-Exos on chondrocytes. Compared to the control group, chondrocyte proliferation was observed at 24, 48, and 72 h after UExo treatment

We conducted a CCK8 assay on human chondrocytes, and the results showed that the cell survival rate in the hUC-MSC-Exos treatment group was higher than that in the control group, with a more significant difference observed over time. The results were similar to those observed in mouse chondrocytes (Fig. 7).

### Safety and efficacy of hUC-MSC-Exos in clinical applications

**Fig. 8:**
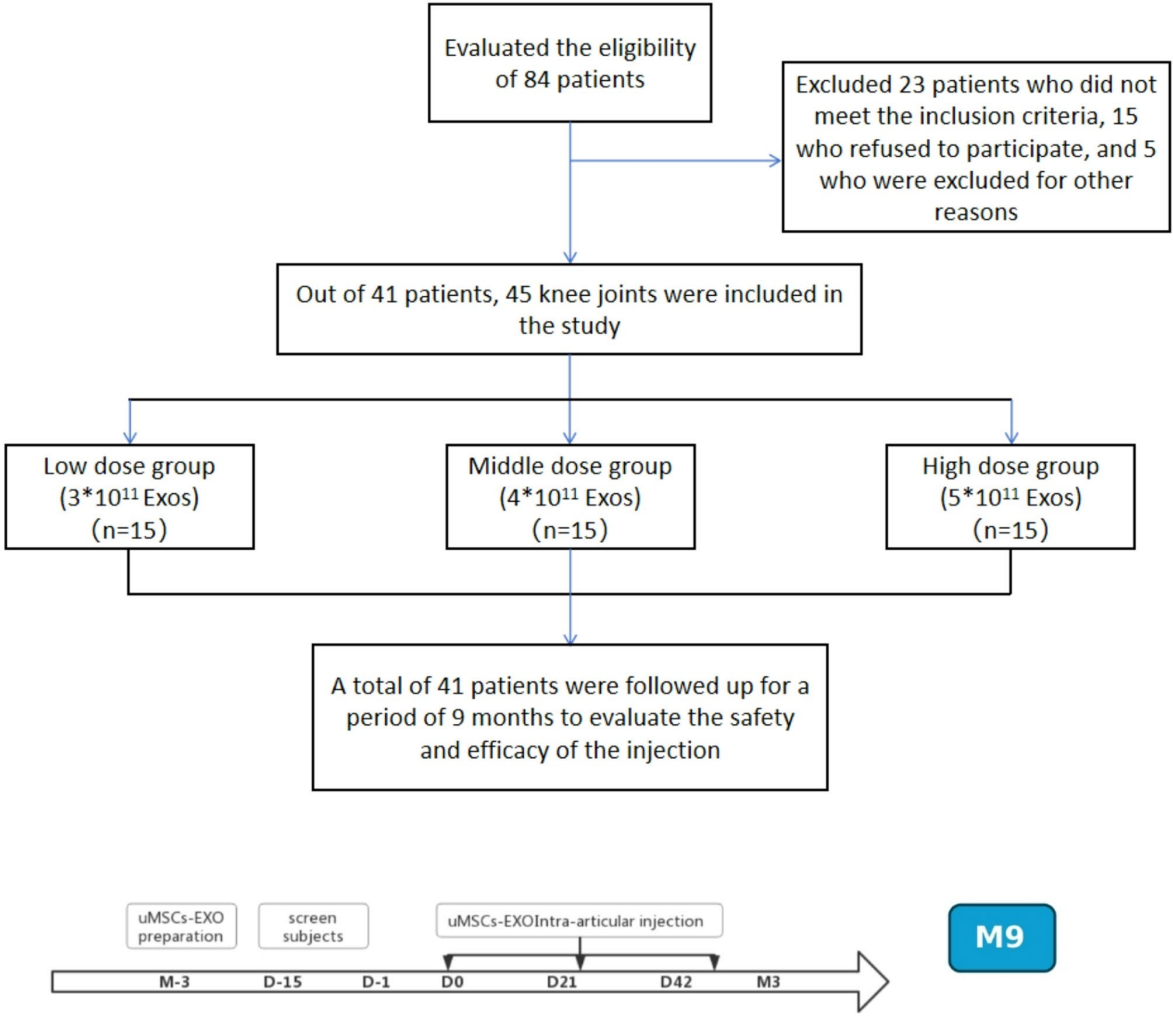
Flowchart of the clinical trial process

Although current research confirms the good efficacy and anti-inflammatory ability of hUC-MSC- Exos in OA, studies such as Tang et al. [51] through in vivo and in vitro preclinical experiments have shown that hUC-MSC-EVs can protect cartilage from damage, with many cartilage repair-related proteins possibly involved in the repair process. Wang et al.‘s [63] study confirmed the anti-inflammatory effect of hUC-MSCs-Exos in human cartilage cell inflammation models. However, these studies are only preclinical. In this study, we conducted a randomized, double-blind, dose-escalation clinical trial to evaluate the clinical safety and efficacy of hUC-MSCs-Exos in treating OA patients. We assessed the eligibility of 84 patients (Fig. 8). Among them, 23 patients who did not meet the inclusion criteria were excluded, 15 patients refused to participate, and 5 patients were not included for other reasons. Overall, 41 patients with 45 knee joints that met the inclusion criteria were assigned to three treatment groups (Fig. 8). There were no sample dropouts during the entire injection process. During the follow-up, one case each in the medium-dose group dropped out at 90 days and 270 days due to undergoing joint replacement surgery and changing contact information, respectively.

The baseline characteristics of patients in each group were similar (Table 2). In general, the average age was 60 years, with a mean body mass index of approximately 26. Despite receiving conservative treatment, all patients had been suffering for more than 5 years. All patients had Kellgren-Lawrence grade 2 or 3 osteoarthritis in the knee joints.

**Table 2:** Baseline characteristics of each group of patients: there are no significant differences in basic information such as age, height, and weight among patients in the high, medium, and low dose groups

Characteristics	low-dose	medium-dose	high-dose
**Age**,** Years**	62.90±0.71	63.26±0.56	63.18±0.61
**Gender**			
male	4(29%)	8(53%)	4(27%)
female	10(71%)	7(47%)	11(73%)
**Height**,** cm**	164±0.36	167±0.30	160±0.50
**Weight**,** kg**	65.5±0.65	70±0.84	67±0.84
**Body-mass index**	24.72±0.21	25.24±0.25	25.33±0.18
**Kellgren-Lawrence grade**			
grade 2	7(15.6%)	7(15.6%)	8(17.8)
grade 3	7(15.6%)	9(20%)	7(15.6%)
**Baseline WOMAC score**	66.5±2.68	82±3.27	88.5±2.11

In the clinical study, despite different doses and varying severity of osteoarthritis among the patient groups, no significant differences were observed in safety during the 9-month follow-up. Physical examinations, laboratory tests, and WOMAC scores showed no significant changes in vital signs or organ system involvement compared to baseline, and there was no increase in WOMAC scores.

**Fig. 9:**
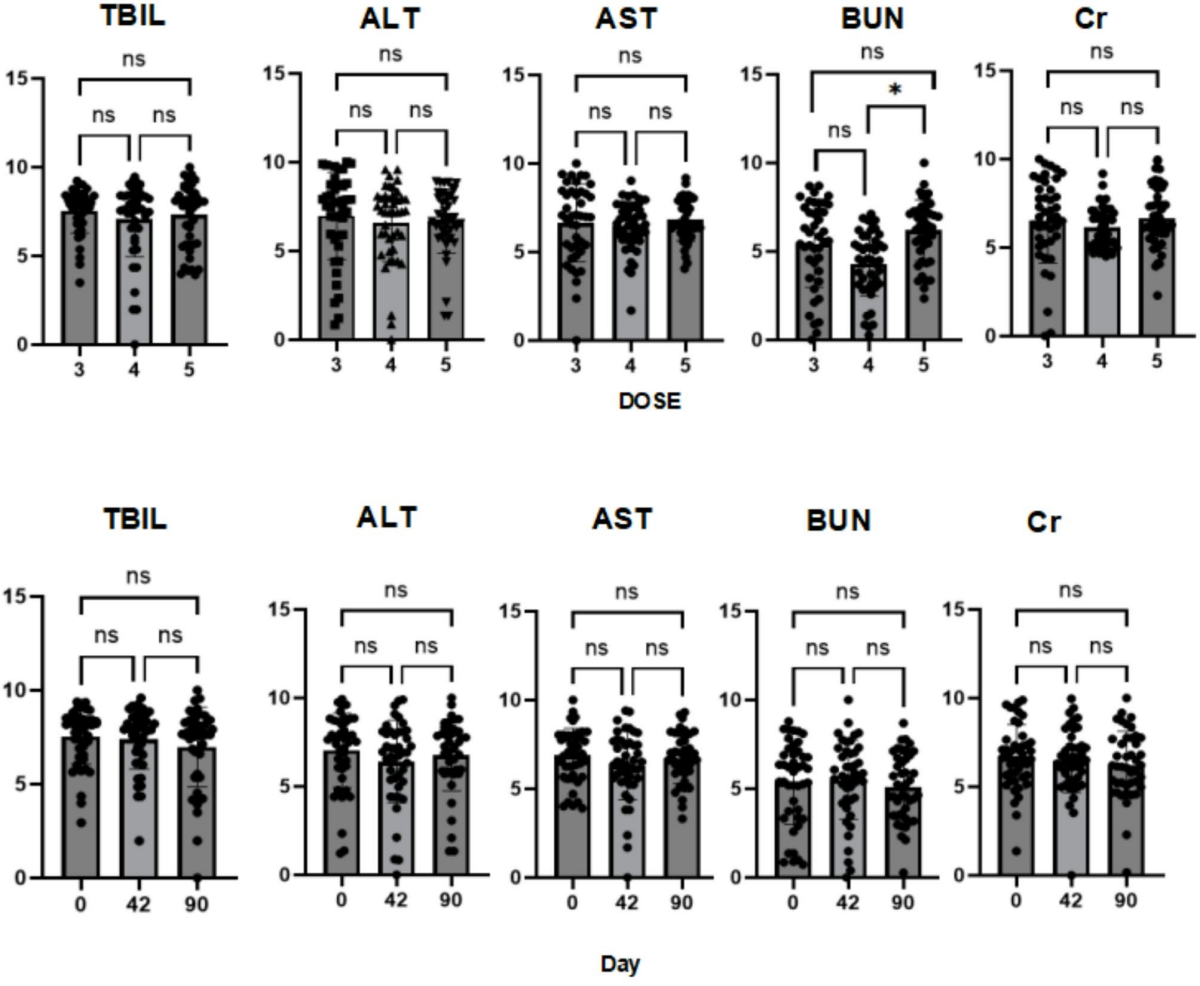
Safety: No significant statistical differences were observed in safety indicators across different injection dose groups and time points

In terms of safety (Tables 3 and 4; Fig. 9), we analyzed several indicators including TBIL, ALT, AST, BUN, and Cr, and observed that except for some differences in BUN across different doses, there were no statistical differences in other indicators. Moreover, no significant differences were observed over time, indicating that intra-articular injection of hUC-MSCs-Exos did not have any impact on the safety of the patients.

**Table 3:** Safety: there are no significant statistical differences in safety indicators among different injection dose groups

Dependent Variable	Dosage (I)	Dosage (J)	Mean Difference (I-J)	Standard error	Sig.
TBIL	3	4	0.48	0.374	0.49
	4	5	-0.26	0.414	0.9
	3	5	-0.23	0.329	0.87
ALT	3	4	0.39	0.503	0.824
	4	5	-0.12	0.427	0.99
	3	5	-0.23	0.329	0.87
AST	3	4	0.1975	0.40124	0.947
	4	5	-0.3731	0.27673	0.451
	3	5	0.1756	0.38246	0.956
BUN	3	4	1.1067	0.46774	0.06
	4	5	-1.8810*	0.37683	0
	3	5	0.7744	0.45606	0.256
Cr	3	4	0.4126	0.41162	0.686
	4	5	-0.5374	0.30903	0.237
	3	5	0.1248	0.45328	0.99

Based on the observed mean

The error term is mean square (error) = 3.336

Comparison using Tamhane’s test. *. The mean difference is significant at the 0.05 level

**Table 4:** Safety no significant statistical differences in safety indicators at different times

Dependent Variable	DAY (I)	DAY (J)	Mean Difference (I-J)	Standard error	Sig.
TBIL	0	42	0.16	0.374	0.674
	42	90	0.42	0.376	0.262
	0	90	0.58	0.376	0.125
ALT	0	42	0.63	0.459	0.169
	42	90	-0.37	0.462	0.428
	0	90	-0.27	0.462	0.563
AST	0	42	0.5833	0.34825	0.096
	42	90	-0.4003	0.35026	0.255
	0	90	-0.183	0.35026	0.602
BUN	0	42	-0.1466	0.45976	0.75
	42	90	0.4118	0.46243	0.375
	0	90	-0.2651	0.46243	0.567
Cr	0	42	0.2347	0.39061	0.549
	42	90	0.2131	0.39288	0.588
	0	90	-0.4479	0.39288	0.256

Based on the observed mean

The error term is mean square (error) = 3.336

Comparison using Tamhane’s test. *. The mean difference is significant at the 0.05 level

In the efficacy analysis, we collected the WOMAC scores of patients before and after treatment and independently compared the pain, stiffness, and difficulty in daily living components. In the analysis at the same follow-up period, no significant differences in efficacy were observed between different dose groups. However, in comparisons within the same dose group, exciting results were observed as the treatment duration increased.

**Fig. 10:**
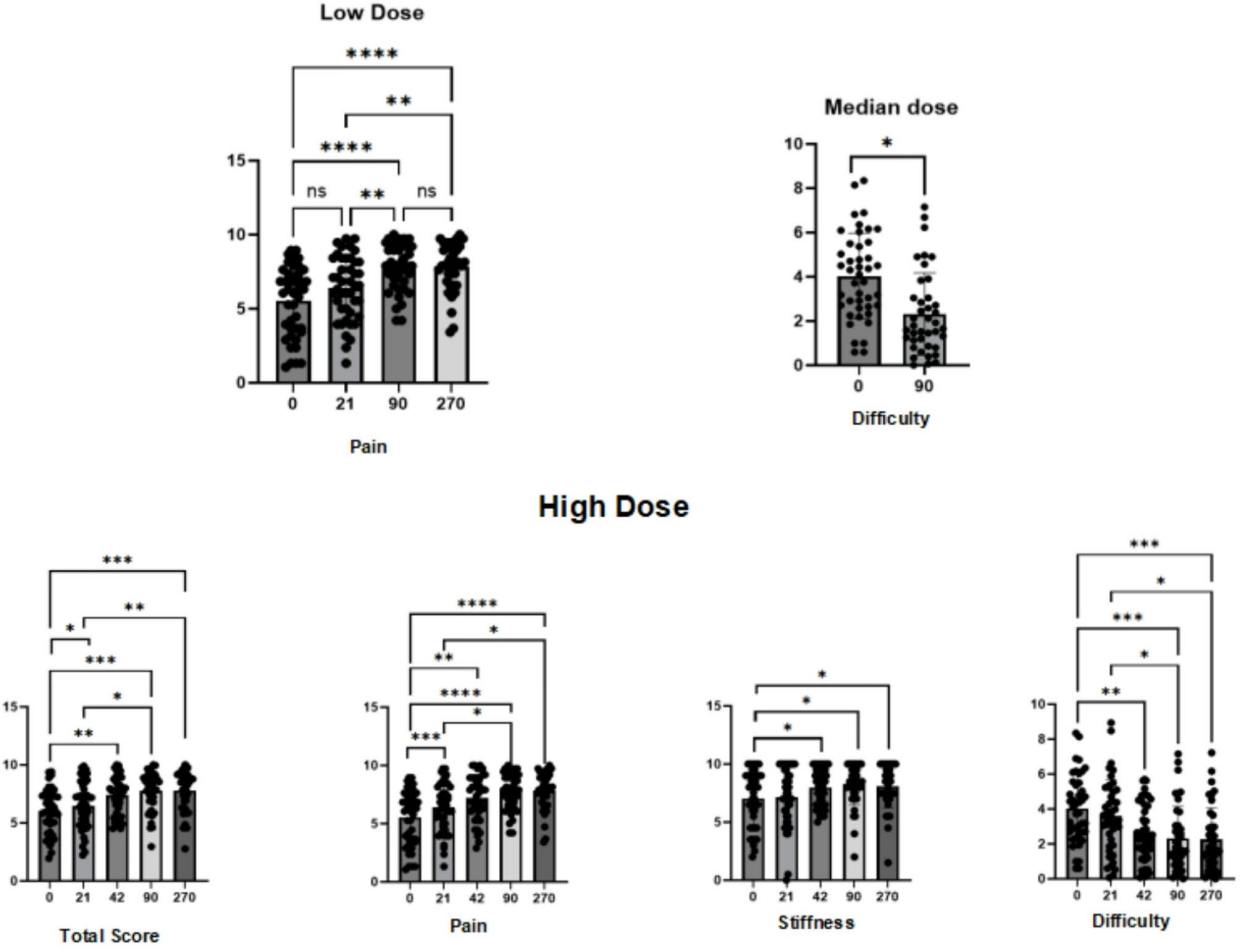
Efficacy analysis at different follow-up times in low, medium, and high dose groups

In the low-dose group (3 × 10^11^), statistically significant differences were found only in the pain score comparison, with the most significant effect observed at 90 days post-treatment (Table 5; Fig. 10).

**Table 5:** Efficacy analysis of the low-dose group at different follow-up times: statistical differences exist only in the improvement of pain scores multiple comparisons of different injection volumes

Dependent Variable	DAY (I)	DAY (J)	Mean Difference (I-J)	Standard Error	Sig.
Pain	0	90	-1.52*	0.624	0.017
	21	90	-1.33*	0.624	0.036
	21	270	-1.28*	0.624	0.044
	0	270	-1.74*	0.624	0.022

Based on the observed mean

The error term is mean square(Error) = 4.067

Comparison using Tamhane’s test. *. The mean difference is significant at the 0.05 level

In the mid-dose group (4 × 10^11^), a statistical difference was observed only in the difficulty in daily living score, with the most significant effect at 90 days post-treatment (Table 6; Fig. 10).

**Table 6:** Efficacy analysis of the medium-dose group at different follow-up times: statistical differences exist only in the improvement of the difficulty of living score

Dependent Variable	DAY (I)	DAY (J)	Mean Difference (I-J)	Standard Error	Sig.
Difficulty in Daily Life	0	90	1.72*	0.854	0.049

Based on the observed mean

The error term is mean square (error) = 4.708

Comparison using Tamhane’s test. *. The mean difference is significant at the 0.05 level

In the high-dose group (5 × 10^11^), statistical differences were observed in the total score, pain, stiffness, and difficulty in daily living, with more significant improvements in the early stages. As the treatment duration increased, the significance of the differences gradually diminished (Table 7; Fig. 10).

**Table 7:** Efficacy analysis at different follow-up times in the high-dose group: there are statistically significant differences in the improvement of total score, pain, stiffness, and difficulty in daily living scores

Dependent Variable	(I) DAY	(J) DAY	Mean Difference (I-J)	Standard Error	Sig.
Total Score	0	21	-1.17*	0.531	0.03
		42	-1.98*	0.531	0
		90	-2.43*	0.54	0
		270	-2.52*	0.54	0
	21	90	-1.25*	0.54	0.023
		270	-1.34*	0.54	0.015
Pain	0	21	-2.09*	0.689	0.003
		42	-3.05*	0.689	0
		90	-3.49*	0.701	0
		270	-3.69*	0.701	0
	21	90	-1.40*	0.701	0.05
		270	-1.61*	0.701	0.025
Stiffness	0	42	-1.50*	0.673	0.029
		90	-1.72*	0.685	0.015
		270	-1.82*	0.685	0.01
Difficulty in Daily Life	0	42	1.74*	0.512	0.001
		90	2.21*	0.521	0
		270	2.27*	0.521	0
	21	90	1.26*	0.521	0.018
		270	1.32*	0.521	0.014

Based on the observed mean

The error term is mean square(Error) = 1.965

Comparison using Tamhane’s test. *. The mean difference is significant at the 0.05 level

In terms of efficacy assessment, hUC-MSCs-Exos showed significant improvements in pain, stiffness, and total scores, with the effects becoming more pronounced as the treatment duration increased. Although no significant statistical differences were found between different dose groups at the same time point, in the comparison of the same dose group at different times, the high-dose group showed the most significant differences. Early improvements in total score and pain were observed at 21 days, while stiffness and difficulty in daily activities showed significant differences only after 42 days. Our analysis suggests that in the early stage of hUC-MSCs-Exos treatment, there is a noticeable reduction in inflammatory responses, and as the treatment progresses, it may contribute to some extent in repairing cartilage tissue and improving quality of life.

**Fig. 11:**
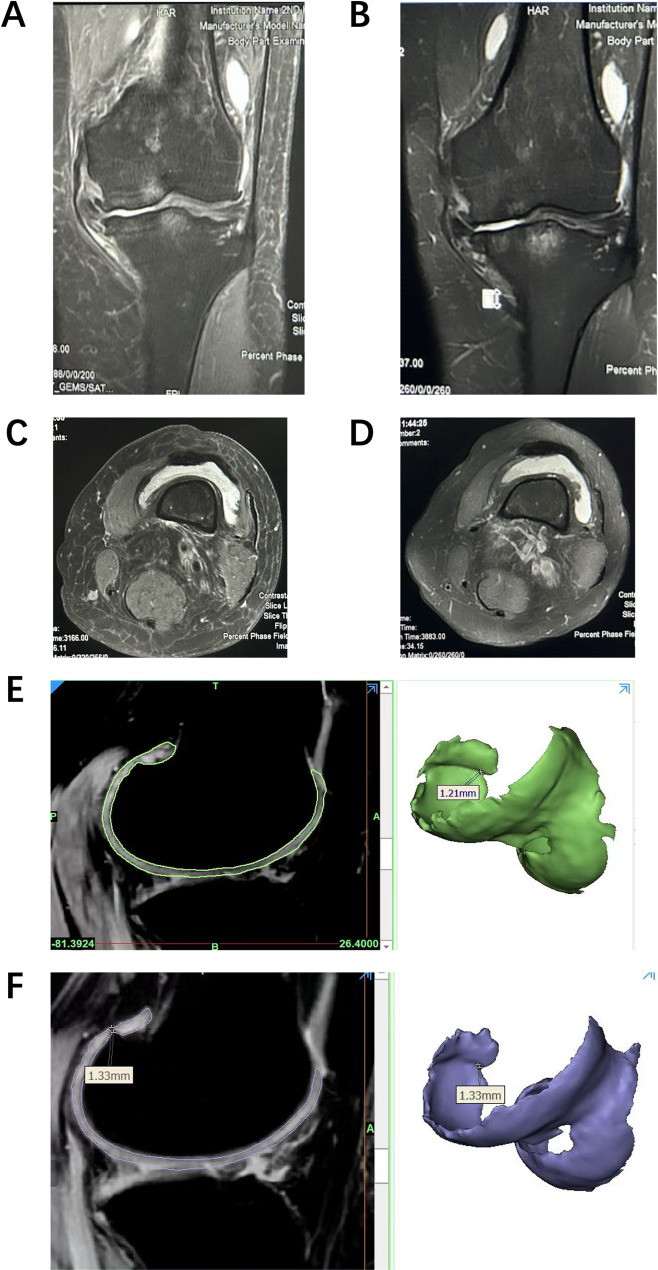
Knee joint MRI before and after hUC-MSCs-Exos injection: (**a**) Pre-injection knee MRI showing extensive bone marrow edema. (**b**) Nine months after injection, knee MRI shows reduced bone marrow edema area. (**c**) Pre-injection knee MRI showing a large volume of joint effusion. (**d**) Post-injection knee MRI showing a smaller volume of joint effusion. (**e**) Pre-injection knee MRI showing significant damage to the femoral cartilage. (**f**) Post-injection knee MRI showing thickened cartilage at the same location of the femoral condyle

Before and after the injection of hUC-MSCs-Exos, MRI scans were performed for some patients, and imaging of cartilage tissue was extracted. It was observed that after the injection of Exos, the knee joint edema was significantly reduced, joint effusion decreased, and the level of knee joint inflammation showed clear improvement (Fig. 11: a\b\c\d). In the cartilage imaging, a certain degree of cartilage thickening was also detected (Fig. 11: e\f). These results indicate that exosome injection may help alleviate or even reverse the progression of OA, offering advantages over other traditional treatment options.

To the best of our knowledge, this is the first clinical application of hUC-MSCs-Exos in the treatment of OA. A clinical trial by Emadedin et al. reported the safety and efficacy of intra-articular injection of hUC-MSCs for treating knee OA [64]. A study by Pers et al. reported the design of a treatment using adipose mesenchymal stem stromal cells for severe knee OA [65]. In our experiment, Exos were injected every 21 days, and hUC-MSCs-Exos maintained long-term improvement in symptom relief, function, and quality of life. After the treatment, patients showed substantial improvements in knee joint function, pain, and quality of life. Combining these results, on one hand, we must acknowledge that, as a cell-derived therapeutic agent, Exos have many advantages compared to whole stem cells: first, Exos have a lower risk than cell transplantation, avoiding immune rejection and carcinogenicity, while providing high stability [66, 67]. Second, the small particles with a lipid bilayer membrane structure make them easier to store and produce. Additionally, Exos can be modified to optimize their targeting ability, therapeutic efficacy, and drug delivery capacity [15, 16]. On the other hand, the disadvantages of Exos therapy should not be overlooked, such as the additional pain caused by multiple injections and the high cost of isolation methods [68]. Objectively, these are the main challenges that need to be overcome in the future.

### The strengths and limitations of this study

In our study, hUC-MSCs-Exos were successfully extracted, and animal experiments were conducted in mice, followed by experiments on human chondrocytes and in vivo studies. Our research provides a relatively comprehensive explanation of the significance of hUC-MSCs-Exos in cartilage repair and OA treatment. Similar studies have not been reported, especially in clinical applications.

Although our study provides some evidence for the clinical application of hUC-MSCs-Exos, there are still several limitations. First, due to funding limitations, the sample size in both preclinical and clinical experiments was relatively small, which may introduce experimental errors, although the results reflect the treatment trend. Additionally, since this is a pilot clinical study mainly assessing the safety of hUC-MSCs-Exos in OA and preliminarily exploring its therapeutic potential, we did not establish a control group due to ethical review considerations. Secondly, the effects of hUC-MSCs-Exos in severe OA patients are still unclear, and most patients with K-L grade 4 OA require surgical treatment. Therefore, the patients included in this study were all below K-L grade 4, and further studies are needed to explore the effects of hUC-MSCs-Exos in severe OA patients. Lastly, due to funding issues, we were unable to perform MRI re-examinations for all patients at 9 months, so we only observed the imaging changes in the cartilage of a portion of the enrolled patients, without conducting in-depth studies on the cellular and molecular mechanisms of its efficacy.

In future studies, we need to secure more funding to conduct in-depth research on the micro-mechanisms of the efficacy of hUC-MSCs-Exos in treating OA and carry out multi-center, large-sample randomized controlled trials (RCTs) to further confirm the optimal dose of therapeutic hUC-MSCs-Exos based on specific conditions or diseases, as well as to compare imaging changes before and after treatment with more samples.

## Conclusion

In this study, we successfully extracted hUC-MSCs and verified their multi-lineage differentiation ability in different culture media. We then confirmed the morphology of Exos and the expression of CD9, CD63, TSG101, and CALN. In both in vitro and in vivo preclinical experiments, we found that hUC-MSC-Exos could potentially reduce the inflammatory response in joint cartilage and promote its regeneration. Finally, no adverse outcomes were observed in the clinical trial involving the injection of hUC-MSC-Exos in OA patients. Moreover, comparisons of clinical scores and MRI scans before and after treatment showed a certain degree of efficacy. Therefore, based on our research findings, hUC-MSC-Exos may provide a promising new therapeutic option for early-stage osteoarthritis in clinical practice.

## Experiments

### Isolation and identification of hUC-MSCs-Exos

#### Extraction of human umbilical cord blood samples

The research protocol using human samples was approved by the Ethics Committee of the Second Hospital of Hebei Medical University and complies with the ethical standards of the Declaration of Helsinki and its subsequent amendments. Before sample collection, all participants were provided with and signed written informed consent. Umbilical cords were obtained from full-term mothers’ placentas, cleaned, and observed under a light microscope.

#### Cell isolation and identification

Cell morphology was observed under a microscope, and cells in the logarithmic growth phase were selected for flow cytometry analysis. Routine digestion was performed using a centrifuge, and cells were resuspended in phosphate-buffered saline (PBS). A volume of 200 µl was added to 1.5 ml EP tubes, with no fewer than 1 × 10^6^ cells per tube. Fluorescent antibodies (from American Biological Reagents) were added to each tube, except for the blank tube, at a concentration of 2 µl. Cells were incubated at room temperature in the dark for 30 min, washed three times with PBS, centrifuged at 800 rpm for 5 minutes, and resuspended in 300 µl PBS. Flow cytometry (Beckman Coulter, Fullerton, CA) was used for detection [69]. UC-MSCs were seeded in a 12-well plate at a density of 1 × 10^5^ cells per well. We followed the experimental method by Hao Wang et al. for hUC-MSCs [70]. For the tri-lineage differentiation experiment, when the cell confluence reached 90–95%, we added adipogenic medium, osteogenic medium, and chondrogenic medium for induction. After 7 days, total RNA was extracted from each group of cells, and real-time quantitative PCR (QPCR) was performed. The expression levels of adipogenic, osteogenic, and chondrogenic genes were analyzed using real-time fluorescence quantitative PCR, with Gapdh as the reference standard. After 28 days of induction, we performed Oil Red O, Alizarin Red, and Alcian Blue staining to observe adipogenesis, osteogenesis, and chondrogenesis.

#### Exosome extraction and identification

HUC-MSCs were seeded onto microcarriers (Cell Ecological Biotechnology Co., Beijing, China) and grown in a three-dimensional suspension using a bioreactor. The medium was refreshed every two days. The resulting culture supernatant was sequentially centrifuged at 300×g for 5 min, 2000×g for 15 min, and 10,000×g for 20 min at 4 °C. The supernatant was filtered through a 0.22 μm PES membrane and then collected and stored at -80 °C.

The pretreated cell culture supernatant was pumped into a tangential flow filtration (TFF) system with hollow fiber columns for circulation and concentration. This continued until the volume was reduced to 1/10 − 1/25 of the original. The concentrated supernatant was then collected into centrifuge tubes. After adding an appropriate amount of cell supernatant exosome extraction reagent (PEG-based) according to the volume, it was mixed thoroughly and precipitated at 4 °C for 2 h. Centrifugation at 10,000×g at 4 °C for 1 h was performed, and the supernatant was separated. The centrifuged supernatant was discarded, and the pellet was resuspended. After mixing, the suspension was stored at -80 °C for further experiments.

Exos (10 µl) were dropped onto a 300-mesh copper grid, stained with 3% phosphotungstic acid solution for 1 min, dried, and observed under a transmission electron microscope (HT7830, HITACHI, Ltd., Tokyo, Japan). Western blot analysis was performed to detect the expression of exosome markers CD63 (ab59479, 1:5000, Abcam), TSG101 (ab125011, 1:5000, Abcam), CD9 (ab92726, 1:5000, Abcam), and Calnexin (ab133615, 1:5000, Abcam). Nanoparticle Tracking Analysis (NTA) was used to determine the size and concentration of Exos.

### In vitro animal experiments

#### Primary chondrocyte culture

Mouse knee joint cartilage tissue samples were extracted and transferred to a cell culture dish, washed with physiological saline containing 2% double antibiotics, then cut into small pieces and transferred to a 50 mL centrifuge tube. The standard laboratory procedure was then followed. The cells were digested with collagenase I (Gibco) at a concentration of 3 mg/mL. The digestion was performed in a shaking incubator at 37 °C for 60–80 min, after which digestion was terminated. The cells were then resuspended in chondrocyte culture medium, seeded into culture flasks, and incubated at 37 °C in a 5% CO2 incubator. The cells were cultured in chondrocyte culture medium until they reached 80–90% confluence, at which point they were passaged. All subsequent experiments were performed using chondrocytes at passages P3 to P5.

#### Cytotoxicity assay of hUC-MSC-Exos

3000 chondrocytes were seeded in each well of a 96-well plate and incubated at 37 °C with 5% CO2 for 24 h. Exos at concentrations of 0 µg/ml and 100 µg/ml were added to the culture medium, with 100 µl added per well. The plates were incubated at 37 °C with 5% CO2 for 24, 48, and 72 h. Afterward, CCK8 reagent was added, and the plates were incubated for 2 h. The absorbance at 450 nm was measured using a microplate reader.

#### The uptake of exos by chondrocytes

According to the instructions provided by the manufacturer (Sigma, USA), Exos were labeled using the cell membrane green fluorescent dye kit DIO. The labeled Exos were then stored at 4 °C for later use. After seeding and attachment of chondrocytes to the wells, the medium containing 100 µg/ml DIO-labeled Exos was replaced, and co-culture was carried out for 1, 5, and 7 days. After incubation, the cells were examined and imaged using a fluorescence microscope.

#### The expression of COL2A1, MMP13, and IL-6 genes in chondrocytes

Chondrocytes were seeded into 12-well plates at a density of 7 × 10⁴ cells per well. Subsequently, 10 ng/mL of IL-1β and a combination of 10 ng/mL IL-1β and 100 mg/mL Exosome were added to each well for 24 h. An equal volume of PBS was used to establish the control group. RNA was extracted from each group and reverse transcribed. Then, QPCR was performed using a real-time fluorescence quantitative system to amplify the expression of COL2A1, MMP13, and IL-6 genes. The system was also used to analyze the expression of the aforementioned genes.

### Study on the efficacy of exosome injection therapy in mouse OA model

#### Mouse OA model and reagent treatment

Three-month-old C57BL/6JNifdc mice were obtained from the Experimental Animal Research Center of Beijing Viton Lihua Laboratory Animal Technology Co., Ltd. (Beijing, China). The mice were randomly divided into three groups: the Normal group (Normal + Veh, *n* = 4), the Model group (OA + Veh, *n* = 9), and the Treatment group (OA + Dexo, *n* = 9). The OA model was established in the Model group by intra-articular injection of a chemical agent. After receiving treatment, the mice were acclimatized for two weeks. On day 0, the mice were anesthetized by intraperitoneal injection of 5% chloral hydrate (200 µL/30 g). The Normal group received 10 µL of physiological saline via intra-articular injection in both knees, while the other two groups received 10 µL of 10% MIA (monosodium iodoacetate) via intra-articular injection once a week for 3 consecutive weeks. The experiment began on the fifth week, and the Treatment group received 10 µL of Uexo (particle count 10^8) via intra-articular injection once a week for 3 consecutive weeks, while the Normal and Model groups received 10 µL of physiological saline. After 4 weeks, a two-week observation period was conducted, and samples were collected for subsequent analysis.

#### Histology

At the end of the experiment, the mice were euthanized, and the entire knee joints were extracted and fixed in 10% formalin for 24 h. Then, the knee joints were treated with 10% EDTA decalcifying solution at 37 °C for 2 to 3 weeks, with the solution changed every three days. After dehydration, the tissues were embedded in paraffin, and 5-µm thick sections were created along the sagittal plane. These sections were stained with Safranin O/Fast Green. Two individuals, who were unaware of the study hypothesis, examined each specimen and assessed the level of cartilage damage using the OARSI cartilage histopathological grading system.

#### IHC analysis

Immunohistochemical staining was performed to detect MMP13 and COL2A1. The sections were deparaffinized, washed with PBS, and subjected to antigen retrieval. Then, 3% BSA was used for blocking at room temperature for 30 min. The primary antibodies were applied to the sections and incubated overnight at 4 °C: COL2 (1:100, 2221110079) and MMP13 (Proteintech, 98899). The sections were then stained with biotinylated secondary antibodies, followed by incubation with streptavidin-horseradish peroxidase solution for visualization.

### Human chondrocyte experiment

#### Preparation of primary chondrocyte cultures

Knee cartilage tissue specimens from clinical knee replacement surgery patients were extracted and transferred to cell culture dishes. The tissue was washed with PBS containing 2% antibiotics, cut into pieces, and transferred to a 50 mL centrifuge tube. Standard laboratory procedures were then followed. The cells were digested with collagenase I (Gibco) at a concentration of 3 mg/mL. Digestion was carried out in a 37 °C shaker for 60–80 min. After digestion, the cells were resuspended in chondrocyte culture medium, seeded into culture flasks, and incubated at 37 °C with 5% CO2. The cells were resuspended in chondrocyte culture medium, seeded into culture flasks, and cultured at 37 °C with 5% CO2 until they reached 80–90% confluence. Passage 3 to 5 chondrocytes were used for all subsequent experiments.

#### Cell toxicity assay of hUC-MSC-Exos

3,000 chondrocytes were seeded in each well of a 96-well plate and incubated at 37 °C with 5% CO2 for 24 h. Exos at concentrations of 0 µg/ml and 100 µg/ml were added to the culture medium at a volume of 100 µl/well, and the plate was incubated at 37 °C with 5% CO2 for 24, 48, and 72 h. Then, CCK8 medium was added, and after 2 h of incubation, the absorbance at 450 nm in each well was measured using a microplate reader.

### The safety and efficacy of intra-articular exosome injection in the treatment of knee osteoarthritis patients

#### Experimental design

The objective of this part of the experiment is to evaluate the safety and efficacy of intra-articular injection of Exos derived from hUC-MSC-Exos for the treatment of knee osteoarthritis patients. A prospective, double-blind, randomized clinical trial was conducted, approved by the Ethics Committee of the Second Hospital of Hebei Medical University (approval number: 2022-T008) and registered on the Chinese National Clinical Trial website ClinicalTrials.gov (trial number: MR-13-24-017929).

#### Sample grouping and observation indicators

Patients were enrolled at the Second Hospital of Hebei Medical University from February 2023 to April 2023. A total of 41 patients with 45 affected limbs, diagnosed with mild to moderate OA of the knee, exhibiting symptoms of pain or swelling for at least 3 months, were included. Detailed inclusion and exclusion criteria are provided in the supplementary materials. All patients were randomly assigned into three groups based on the K-L grade: low-dose (3*10^*11*^*exosome particles)*, medium-dose (4*10^11^ exosome particles), and high-dose (5*10^11^ exosome particles), with 15 patients in each group. They received a 2.5 mL exosome particle solution injection into the joint cavity on days 0, 21, and 42. All injections were performed by a specialist physician in an outpatient setting, with both the physician and patients blinded to the dose of hUC-MSC-Exos injections.

Observation indicators: (1) Blood tests for routine blood work, liver, and kidney function were conducted before each injection, immediately after each injection, and 3 months after enrollment to assess the safety of exosome injections. (2) WOMAC (Western Ontario and McMaster Universities Osteoarthritis Index) scores were recorded before each injection, immediately after each injection, and during follow-up at 3, 6, and 9 months to assess clinical outcomes. (3) MRI of the knee joint was performed at baseline and at 9 months post-enrollment to evaluate the effectiveness of treatment and cartilage regeneration.

#### Randomization and blinding

OA and K-L grades were determined using standard radiographic examination OA and K-L grades were determined using standard radiographic examination [71], assessed by experienced orthopedic surgeons or trained musculoskeletal radiologists. We collected demographic information and baseline characteristics. All patients underwent clinical examinations (routine knee joint examination, WOANC scoring, MRI scans, etc., detailed in Table 2), which were completed by rheumatologists or orthopedic doctors before the trial began or during the 3-month follow-up period.

Patients were randomly assigned to receive joint cavity injections of Exos at three different doses. Randomization was carried out using group randomization with a group size of 41 patients, where four patients received bilateral knee joint injections, requiring two rounds of randomization. Each randomization group consisted of 45 opaque envelopes, each containing a card with the drug name written on it (15 cards for low dose, 15 for medium dose, 15 for high dose). The envelopes were stored in a locked safe. Before the first injection, the drug provider retrieved an envelope from the safe to determine the patient’s treatment group. During subsequent injections, both the injecting physician and the patient remained blinded to the injection dose.

For patients with bilateral knee joints, they were randomly assigned with an interval of at least 3 months between injections in each knee. All patients and researchers, except for the drug provider, were blinded. All exosome doses were stored in identical antibiotic vials, and the drugs had the same color and volume to avoid distinguishing between doses. A research nurse registered the patients and checked all inclusion and exclusion criteria with the patients. If any criteria were unclear, the primary investigator (MT) or sponsor investigator (ABR) was consulted.

#### Intervention

On the day of injection, both the researcher and the subject received the quality control report of the study drug. The researcher checked the production date, dose, storage conditions, expiration date, and subject-related information before clinical use to ensure accuracy. Subjects confirmed the safety of the clinical trial product and underwent regular check-ups as per the study protocol to observe the efficacy and safety of exosome treatment.

After production, the exosome injection was delivered to the hospital through low-temperature transportation. There was no drug return during the study, and all operations were conducted under strict sterile conditions. We sent surveys before each injection and again at 3 months, 6 months, and 9 months after the intervention. After 9 months, a random subset of patients from all three groups was selected for MRI examination. The primary outcome for pain assessment within 9 months included the WOMAC pain component (range, 0–20), WOMAC stiffness component (range, 0–8), and physical function (range, 0–68), with all adverse events recorded.

### Statistical analysis

Statistical analysis was performed by three professional statisticians using GraphPad Prism 8.0.2 for preclinical research data, conducting statistical analysis via one-way ANOVA and Tukey’s post-hoc test. Clinical trial data were analyzed using SPSS 26. All data were quantified, with the maximum score set to 10. Mean differences (I-J) were recorded, and normality was assessed through variance homogeneity analysis. If variances were homogeneous, LSD was used for statistical analysis; if variances were heterogeneous, the Tamhane method was employed. A p-value < 0.05 was considered statistically significant for all comparisons.

## Electronic supplementary material

Below is the link to the electronic supplementary material.

Supplementary Material 1

## Data Availability

All the data and materials were presented in the main paper.

## Abbreviations

OA: Osteoarthritis
MSCs: Mesenchymal stem cells
Exos: Exosomes
IL-6: Interleukin 6
COL2A1: Type II collagen
hUC-MSCs: Human umbilical cord mesenchymal stem cells
UMSC: Umbilical cord-derived mesenchymal stem cells
ROS: Reactive oxygen species
ECM: Extracellular matrix
OARSI: Osteoarthritis Research Society International
hUC-MSC-EVs: Human umbilical cord mesenchymal stem cells-derived extracellular vesicles
RT-qPCR: Real-time quantitative polymerase chain reaction
TFF: Tangential flow filtration
NTA: Nanoparticle tracking analysis
uMSCs-EXO: Umbilical Mesenchymal Stem Cell-derived Exosomes
MMP13: Metalloproteinase 13
WOMAC: Western Ontario and McMaster University Osteoarthritis Index
ACR: American College of Rheumatology
NRS: Numerical rating scale
BMI: Body mass index

## References

[1] Glyn-Jones S, Palmer AJR, Agricola R, Price AJ, Vincent TL, Weinans H, et al. Osteoarthr Lancet. 2015;386:376–87. 10.1016/S0140-6736(14)60802-3 25748615

[2] Kotlarz H, Gunnarsson CL, Fang H, Rizzo JA. Insurer and out-of-pocket costs of osteoarthritis in the US: evidence from National survey data. Arthritis Rheum. 2009;60:3546–53. 19950287 10.1002/art.24984

[3] James SL, Abate D, Abate KH, Abay SM, Abbafati C, Abbasi N et al. Global, regional, and national incidence, prevalence, and years lived with disability for 354 diseases and injuries for 195 countries and territories, 1990–2017: a systematic analysis for the Global Burden of Disease Study 2017. The Lancet [Internet]. 2018;392:1789–858. Available from: https://pubmed.ncbi.nlm.nih.gov/30496104/10.1016/S0140-6736(18)32279-7 PMC6227754 30496104

[4] Escobar Ivirico JL, Bhattacharjee M, Kuyinu E, Nair LS, Laurencin CT. Regenerative engineering for knee osteoarthritis treatment: biomaterials and Cell-Based technologies. Engineering. 2017;3:16–27. 35392109 10.1016/j.eng.2017.01.003 PMC8986132

[5] Bannuru RR, Osani MC, Vaysbrot EE, Arden NK, Bennell K, Bierma-Zeinstra SMA, et al. OARSI guidelines for the non-surgical management of knee, hip, and polyarticular osteoarthritis. Osteoarthritis Cartilage. 2019;27:1578–89. 31278997 10.1016/j.joca.2019.06.011

[6] LIM DIEPPEP. Who should have knee joint replacement surgery for osteoarthritis? Int J Rheum Dis. 2011;14:175–80. 21518317 10.1111/j.1756-185X.2011.01611.x

[7] Richard MJ, Driban JB, McAlindon TE. Pharmaceutical treatment of osteoarthritis. Osteoarthritis Cartilage. 2022;31:458–66. 36414224 10.1016/j.joca.2022.11.005

[8] Madry H. Surgical therapy in osteoarthritis. Osteoarthritis Cartilage. 2022;30:1019–34. 35183776 10.1016/j.joca.2022.01.012

[9] Akbari A, Jabbari N, Sharifi R, Ahmadi M, Vahhabi A, Seyedzadeh SJ, et al. Free and hydrogel encapsulated exosome-based therapies in regenerative medicine. Life Sci. 2020;249:117447. 32087234 10.1016/j.lfs.2020.117447

[10] Chang YH, Wu KC, Harn HJ, Lin SZ, Ding DC. Exos and stem cells in degenerative disease diagnosis and therapy. Cell Transpl. 2018;27:349–63. 10.1177/0963689717723636 PMC6038041 29692195

[11] Meldolesi J. Exos and ectosomes in intercellular communication. Curr Biol. 2018;28:R435–44. 29689228 10.1016/j.cub.2018.01.059

[12] Nikfarjam S, Rezaie J, Zolbanin NM, Jafari R. Mesenchymal stem cell derived-Exos: a modern approach in translational medicine. J Transl Med. 2020;18:449. 33246476 10.1186/s12967-020-02622-3 PMC7691969

[13] He L, Chen Y, Ke Z, Pang M, Yang B, Feng F, et al. Exos derived from miRNA-210 overexpressing bone marrow mesenchymal stem cells protect lipopolysaccharide induced chondrocytes injury via the NF-κB pathway. Gene. 2020;751:144764. 32428694 10.1016/j.gene.2020.144764

[14] Wang Z, Yan K, Ge G, Zhang D, Bai J, Guo X, et al. Exos derived from miR-155-5p–overexpressing synovial mesenchymal stem cells prevent osteoarthritis via enhancing proliferation and migration, attenuating apoptosis, and modulating extracellular matrix secretion in chondrocytes. Cell Biol Toxicol. 2020;37:85–96. 33099657 10.1007/s10565-020-09559-9

[15] Zhou L, Ye H, Liu L, Chen Y. Human Bone Mesenchymal Stem Cell-Derived Exos Inhibit IL-1β-Induced Inflammation in Osteoarthritis Chondrocytes. Cell J [Internet]. 2021;23:485–94. Available from: https://pubmed.ncbi.nlm.nih.gov/34455725/10.22074/cellj.2021.7127 PMC8405079 34455725

[16] Xu H, Xu B. BMSC-Derived exos ameliorate osteoarthritis by inhibiting pyroptosis of cartilage via delivering miR-326 targeting HDAC3 and STAT1//NF-κB p65 to chondrocytes. Yokota S Ichi. Editor Mediators Inflamm. 2021;2021:1–26. 10.1155/2021/9972805 PMC8577926 34764819

[17] Qi H, Liu DP, Xiao DW, Tian DC, Su YW, Jin SF. Exos derived from mesenchymal stem cells inhibit mitochondrial dysfunction-induced apoptosis of chondrocytes via p38, ERK, and Akt pathways. Vitro Cell Dev Biol Anim. 2019;55:203–10. 10.1007/s11626-019-00330-x 30783864

[18] Zhang Z, Zhao S, Sun Z, Zhai C, Xia J, Wen C, et al. Enhancement of the therapeutic efficacy of mesenchymal stem cell-derived exos in osteoarthritis. Cell Mol Biol Lett. 2023;28:75. 37770821 10.1186/s11658-023-00485-2 PMC10540339

[19] Lopez J, Al-Nakkash L, Broderick TL, Castro M, Tobin B, Plochocki JH. Genistein Suppresses IL-6 and MMP-13 to Attenuate Osteoarthritis in Obese Diabetic Mice. Metabolites [Internet]. 2023;13:1014. Available from: https://pubmed.ncbi.nlm.nih.gov/37755294/10.3390/metabo13091014 PMC10534591 37755294

[20] Nambi G. Does low level laser therapy has effects on inflammatory biomarkers IL-1β, IL-6, TNF-α, and MMP-13 in osteoarthritis of rat models—a systemic review and meta-analysis. Lasers Med Sci. 2020;36:475–84. 32833088 10.1007/s10103-020-03124-w

[21] Yaghoubi Y, Movassaghpour A, Zamani M, Talebi M, Mehdizadeh A, Yousefi M. Human umbilical cord mesenchymal stem cells derived-Exos in diseases treatment. Life Sci. 2019;233:116733. 31394127 10.1016/j.lfs.2019.116733

[22] Amable P, Teixeira MV, Carias RB, Granjeiro J, Borojevic R. Protein synthesis and secretion in human mesenchymal cells derived from bone marrow, adipose tissue and Wharton’s jelly. Stem Cell Res Ther. 2014;5:53. 24739658 10.1186/scrt442 PMC4055160

[23] Kobolak J, Dinnyes A, Memic A, Khademhosseini A, Mobasheri A. Mesenchymal stem cells: identification, phenotypic characterization, biological properties and potential for regenerative medicine through biomaterial micro-engineering of their niche. Methods. 2016;99:62–8. 26384580 10.1016/j.ymeth.2015.09.016

[24] Zhu SF, Zhong ZN, Fu XF, Peng DX, Lu GH, Li WH et al. Comparison of cell proliferation, apoptosis, cellular morphology and ultrastructure between human umbilical cord and placenta-derived mesenchymal stem cells. Neurosci Lett [Internet]. 2013;541:77–82. Available from: https://www.sciencedirect.com/science/article/abs/pii/S0304394013002231?via%3Dihub 10.1016/j.neulet.2013.03.018 23523648

[25] Nagamura-Inoue T. Umbilical cord-derived mesenchymal stem cells: their advantages and potential clinical utility. World J Stem Cells. 2014;6:195–202. 24772246 10.4252/wjsc.v6.i2.195 PMC3999777

[26] Mennan C, Brown S, McCarthy H, Mavrogonatou E, Kletsas D, Garcia J, et al. Mesenchymal stromal cells derived from whole human umbilical cord exhibit similar properties to those derived from Wharton’s jelly and bone marrow. FEBS Open Bio. 2016;6:1054–66. 27833846 10.1002/2211-5463.12104 PMC5095143

[27] Cardoso TC, Okamura LH, Baptistella JC, Gameiro R, Ferreira HL, Marinho M, et al. Isolation, characterization and immunomodulatory-associated gene transcription of Wharton’s jelly-derived multipotent mesenchymal stromal cells at different trimesters of cow pregnancy. Cell Tissue Res. 2016;367:243–56. 27677269 10.1007/s00441-016-2504-9

[28] Esteves CL, Sheldrake TA, Mesquita SP, Pesántez JJ, Menghini T, Dawson L, et al. Isolation and characterization of equine native MSC populations. Stem Cell Res Ther. 2017;8:80. 28420427 10.1186/s13287-017-0525-2 PMC5395828

[29] Burk J, Holland H, Lauermann AF, May T, Siedlaczek P, Charwat V, et al. Generation and characterization of a functional human adipose-derived multipotent mesenchymal stromal cell line. Biotechnol Bioeng. 2019;116:1417–26. 30739319 10.1002/bit.26950

[30] Sturiale S, Barbara G, Qiu B, Figini M, Geppetti P, Gerard N, et al. Neutral endopeptidase (EC 3.4.24.11) terminates colitis by degrading substance P. Proc Natl Acad Sci U S A. 1999;96:11653–8. 10500232 10.1073/pnas.96.20.11653 PMC18089

[31] Tognoli ML, Dancourt J, Bonsergent E, Palmulli R, de Jong OG, Van Niel G et al. Lack of involvement of CD63 and CD9 tetraspanins in the extracellular vesicle content delivery process. Commun Biol [Internet]. 2023;6:1–9. Available from: https://www.nature.com/articles/s42003-023-04911-1 10.1038/s42003-023-04911-1 PMC10192366 37198427

[32] Colombo M, Moita C, van Niel G, Kowal J, Vigneron J, Benaroch P et al. Analysis of ESCRT functions in exosome biogenesis, composition and secretion highlights the heterogeneity of extracellular vesicles. J Cell Sci [Internet]. 2013;126:5553–65. Available from: https://jcs.biologists.org/content/126/24/5553.long 10.1242/jcs.128868 24105262

[33] Giordano C, Gelsomino L, Barone I, Panza S, Augimeri G, Bonofiglio D, et al. Leptin modulates exosome biogenesis in breast Cancer cells: an additional mechanism in Cell-to-Cell communication. J Clin Med. 2019;8:1027. 31336913 10.3390/jcm8071027 PMC6678227

[34] Théry C, Witwer KW, Aikawa E, Alcaraz MJ, Anderson JD, Andriantsitohaina R, et al. Minimal information for studies of extracellular vesicles 2018 (MISEV2018): a position statement of the international society for extracellular vesicles and update of the MISEV2014 guidelines. J Extracell Vesicles. 2018;7:1535750. 30637094 10.1080/20013078.2018.1535750 PMC6322352

[35] Wu Y, Deng W, Klinke DJ. Exos: improved methods to characterize their morphology, RNA content, and surface protein biomarkers. Analyst [Internet]. 2015;140:6631–42. Available from: https://pubmed.ncbi.nlm.nih.gov/26332016/10.1039/c5an00688k PMC4986832 26332016

[36] Kloppenburg M, Berenbaum F. Osteoarthritis year in review 2019: epidemiology and therapy. Osteoarthritis Cartilage. 2020;28:242–8. 31945457 10.1016/j.joca.2020.01.002

[37] Zhao L, Chen S, Yang P, Cao H, Li L. The role of mesenchymal stem cells in hematopoietic stem cell transplantation: prevention and treatment of graft-versus-host disease. Stem Cell Res Ther. 2019;10:182. 31227011 10.1186/s13287-019-1287-9 PMC6588914

[38] de Castro LL, Lopes-Pacheco M, Weiss DJ, Cruz FF, Rocco PRM. Current understanding of the immunosuppressive properties of mesenchymal stromal cells. J Mol Med (Berl) [Internet]. 2019;97:605–18. Available from: https://pubmed.ncbi.nlm.nih.gov/30903229/10.1007/s00109-019-01776-y 30903229

[39] Guillamat-Prats R, Camprubí-Rimblas M, Bringué J, Tantinyà N, Artigas A. Cell therapy for the treatment of sepsis and acute respiratory distress syndrome. Ann Transl Med. 2017;5:446. 29264363 10.21037/atm.2017.08.28 PMC5721220

[40] Lai RC, Yeo RWY, Lim SK. Mesenchymal stem cell exos. Semin Cell Dev Biol. 2015;40:82–8. 25765629 10.1016/j.semcdb.2015.03.001

[41] Kourembanas S, Exos. Vehicles of intercellular signaling, biomarkers, and vectors of cell therapy. Annu Rev Physiol. 2015;77:13–27. 25293529 10.1146/annurev-physiol-021014-071641

[42] Toh WS, Lai RC, Hui JHP, Lim SK. MSC exosome as a cell-free MSC therapy for cartilage regeneration: implications for osteoarthritis treatment. Semin Cell Dev Biol. 2017;67:56–64. 27871993 10.1016/j.semcdb.2016.11.008

[43] Zhang J, Rong Y, Luo C, Cui W. Bone marrow mesenchymal stem cell-derived exos prevent osteoarthritis by regulating synovial macrophage polarization. Aging. 2020;12:25138–52. 33350983 10.18632/aging.104110 PMC7803581

[44] Zhou H, Shen X, Yan C, Xiong W, Tan ZM. Extracellular vesicles derived from human umbilical cord mesenchymal stem cells alleviate osteoarthritis of the knee in mice model by interacting with METTL3 to reduce m6A of NLRP3 in macrophage. Stem Cell Res Ther. 2022;13:322. 35842714 10.1186/s13287-022-03005-9 PMC9288728

[45] Pan T, Wu D, Cai N, Chen R, Shi X, Li B, et al. Alpha-Mangostin protects rat articular chondrocytes against IL-1β-induced inflammation and slows the progression of osteoarthritis in a rat model. Int Immunopharmacol. 2017;52:34–43. 28858724 10.1016/j.intimp.2017.08.010

[46] Primrose JGB, Jain L, Alhilali M, Bolam SM, Monk AP, Munro JT, et al. REST, RCOR1 and RCOR2 expression is reduced in Osteoarthritic chondrocytes and contributes to increasing MMP13 and ADAMTS5 expression through upregulating HES1. Cell Signal. 2023;109:110800. 37442513 10.1016/j.cellsig.2023.110800

[47] Sellam J, Berenbaum F. The role of synovitis in pathophysiology and clinical symptoms of osteoarthritis. Nat Rev Rheumatol [Internet]. 2010;6:625–35. Available from: https://www.nature.com/articles/nrrheum.2010.159 10.1038/nrrheum.2010.159 20924410

[48] Berenbaum F. Osteoarthritis as an inflammatory disease (osteoarthritis is not osteoarthrosis!). Osteoarthritis Cartilage [Internet]. 2013;21:16–21. Available from: https://www.sciencedirect.com/science/article/pii/S1063458412010254 10.1016/j.joca.2012.11.012 23194896

[49] Aigner T, McKenna L. Molecular pathology and pathobiology of Osteoarthritic cartilage. Cell Mol Life Sci. 2002;59:5–18. 11846033 10.1007/s00018-002-8400-3 PMC11337501

[50] Benito MJ. Synovial tissue inflammation in early and late osteoarthritis. Ann Rheum Dis [Internet]. 2005;64:1263–7. Available from: https://www.ncbi.nlm.nih.gov/pmc/articles/PMC1755629/10.1136/ard.2004.025270 PMC1755629 15731292

[51] Tang S, Chen P, Zhang H, Weng H, Fang Z, Chen C et al. Comparison of Curative Effect of Human Umbilical Cord-Derived Mesenchymal Stem Cells and Their Small Extracellular Vesicles in Treating Osteoarthritis. Int J Nanomedicine [Internet]. 2021;16:8185–202. Available from: https://www.ncbi.nlm.nih.gov/pmc/articles/PMC8687685/10.2147/IJN.S336062 PMC8687685 34938076

[52] Jiang S, Tian G, Yang Z, Gao X, Wang F, Li J et al. Enhancement of acellular cartilage matrix scaffold by Wharton’s jelly mesenchymal stem cell-derived Exos to promote osteochondral regeneration. Bioact Mater [Internet]. 2021;6:2711–28. Available from: https://www.sciencedirect.com/science/article/pii/S2452199X21000438?via%3Dihub 10.1016/j.bioactmat.2021.01.031 PMC7895679 33665503

[53] Kouroupis D, Kaplan LD, Best TM. Human infrapatellar fat pad mesenchymal stem cells show Immunomodulatory Exosomal signatures. Sci Rep. 2022;12:S85–6. 10.1038/s41598-022-07569-7 PMC8897449 35246587

[54] Zhang Y, Bai J, Xiao B, Li C. BMSC-derived exos promote osteoporosis alleviation via M2 macrophage polarization. Mol Med. 2024;30:220. 39563244 10.1186/s10020-024-00904-w PMC11577737

[55] Barajas-Gómez BA, Rosas-Carrasco O, Morales-Rosales SL, Pedraza Vázquez G, González-Puertos VY, Juárez-Cedillo T, et al. Relationship of inflammatory profile of elderly patients serum and senescence-associated secretory phenotype with human breast cancer cells proliferation: role of IL6/IL8 ratio. Cytokine. 2017;91:13–29. 27951455 10.1016/j.cyto.2016.12.001

[56] Hu Q, Ecker M. Overview of MMP-13 as a promising target for the treatment of osteoarthritis. Int J Mol Sci. 2021;22:1742. 33572320 10.3390/ijms22041742 PMC7916132

[57] Yao X, Sun K, Yu S, Luo J, Guo J, Lin J et al. Chondrocyte ferroptosis contribute to the progression of osteoarthritis. J Orthop Translat [Internet]. 2021;27:33–43. Available from: https://www.sciencedirect.com/science/article/pii/S2214031X20301108?via%3Dihub 10.1016/j.jot.2020.09.006 PMC7750492 33376672

[58] Bapat S, Hubbard D, Munjal A, Hunter M, Fulzele S. Pros and cons of mouse models for studying osteoarthritis. Clin Transl Med. 2018;7. 10.1186/s40169-018-0215-4 PMC6246759 30460596

[59] Yang CY, Chanalaris A, Troeberg L. ADAMTS and ADAM metalloproteinases in osteoarthritis– looking beyond the ‘usual suspects’. Osteoarthritis Cartilage. 2017;25:1000–9. 28216310 10.1016/j.joca.2017.02.791 PMC5473942

[60] Gerwin N, Bendele AM, Glasson S, Carlson CS. The OARSI histopathology initiative– recommendations for histological assessments of osteoarthritis in the rat. Osteoarthritis Cartilage. 2010;18:S24–34. 20864021 10.1016/j.joca.2010.05.030

[61] Zhu JK, He TD, Wei ZX, Wang YM. LncRNA FAS-AS1 promotes the degradation of extracellular matrix of cartilage in osteoarthritis. Eur Rev Med Pharmacol Sci [Internet]. 2018;22:2966–72. Available from: https://pubmed.ncbi.nlm.nih.gov/29863238/10.26355/eurrev_201805_15051 29863238

[62] Grigull NP, Redeker JI, Schmitt B, Saller MM, Schönitzer V, Mayer-Wagner S. Chondrogenic potential of pellet culture compared to High-Density culture on a bacterial cellulose hydrogel. Int J Mol Sci. 2020;21:2785. 32316353 10.3390/ijms21082785 PMC7215943

[63] Wang S, Jiang W, Lv S, Sun Z, Si L, Hu J et al. Human umbilical cord mesenchymal stem cells-derived Exos exert anti-inflammatory effects on osteoarthritis chondrocytes. Aging [Internet]. 2023;15:9544–60. Available from: https://pubmed.ncbi.nlm.nih.gov/37724890/10.18632/aging.205034 PMC10564422 37724890

[64] LABIBZADEH EMADEDINM, LIASTANI N, KARIMI MG, JAROUGHI A, BOLURIEH N. Intra-articular implantation of autologous bone marrow–derived mesenchymal stromal cells to treat knee osteoarthritis: a randomized, triple-blind, placebo-controlled phase 1/2 clinical trial. Cytotherapy. 2018;20:1238–46. 30318332 10.1016/j.jcyt.2018.08.005

[65] Pers YM, Rackwitz L, Ferreira R, Pullig O, Delfour C, Barry F, et al. Adipose mesenchymal stromal Cell-Based therapy for severe osteoarthritis of the knee: A phase I Dose-Escalation trial. Stem Cells Transl Med. 2016;5:847–56. 27217345 10.5966/sctm.2015-0245 PMC4922848

[66] Wang C, Zhou H, Wu R, Guo Y, Gong L, Fu K et al. Mesenchymal stem cell-derived Exos and non-coding RNAs: Regulatory and therapeutic role in liver diseases. Biomed Pharmacother [Internet]. 2023;157:114040. Available from: https://pubmed.ncbi.nlm.nih.gov/36423545/10.1016/j.biopha.2022.114040 36423545

[67] Lou G, Chen Z, Zheng M, Liu Y. Mesenchymal stem cell-derived exos as a new therapeutic strategy for liver diseases. Exp Mol Med. 2017;49:e346. 28620221 10.1038/emm.2017.63 PMC5519012

[68] Li J, Zhang Y, Dong PY, Yang GM, Gurunathan S. A comprehensive review on the composition, biogenesis, purification, and multifunctional role of exosome as delivery vehicles for cancer therapy. Biomedicine & Pharmacotherapy [Internet]. 2023;165:115087. Available from: https://www.sciencedirect.com/science/article/pii/S0753332223008788 10.1016/j.biopha.2023.115087 37392659

[69] Wang Z, Li X, He X, Wu B, Xu M, Chang H, et al. Delivery of the Sox9 gene promotes chondrogenic differentiation of human umbilical cord blood-derived mesenchymal stem cells in an in vitro model. Braz J Med Biol Res. 2014;47:279–86. 24652327 10.1590/1414-431X20133539 PMC4075291

[70] Wang H, Yan X, Jiang Y, Wang Z, Li Y, Shao Q. The human umbilical cord stem cells improve the viability of OA degenerated chondrocytes. Mol Med Rep. 2018;17:4474–82. 29328479 10.3892/mmr.2018.8413 PMC5802223

[71] Kellgren JH, Lawrence JS. Radiological Assessment of Osteo-Arthrosis. Ann Rheum Dis [Internet]. 1957;16:494–502. Available from: https://www.ncbi.nlm.nih.gov/pmc/articles/PMC1006995/10.1136/ard.16.4.494 PMC1006995 13498604

