# Supplementary material for: Injection of human umbilical cord mesenchymal stem cells exosomes for the treatment of knee osteoarthritis: from preclinical to clinical research
Source: J Transl Med. 2025 Jun 11;23:641. doi: 10.1186/s12967-025-06623-y (PMC12153132; doi:10.1186/s12967-025-06623-y)
Supplement: Supplementary file 1 — Supplementary Material 1 [file 12967_2025_6623_MOESM1_ESM.docx]

**Supplementary Materials**

1. **Inclusion criteria**

**Main inclusion criteria:**

1. Patients diagnosed with knee osteoarthritis grade 1 and 2 based on clinical and radiological criteria of the American College of Rheumatology (ACR) classification criteria.

2. Patients with pain lasting for more than half a year and pain or swelling for at least 3 months, or patients with osteoarthritis recurring or aggravating after stopping conventional clinical treatment, such as hormones, opioids, viscoelastic supplementation, etc.

3. Morning stiffness less than 30 minutes.

4. Kellgren-Lawrence grade of the target study side knee joint is II-III.

5. The score of the Western Ontario and McMaster University WOMAC pain sub-item A1 determined in the 11-point numerical rating scale (NRS) is at least 4 points, and chronic pain lasts for at least 4 weeks when signing the informed consent.

6. 40-80 years old, regardless of gender.

7. Able to understand and voluntarily sign the informed consent form, and voluntarily complete the trial procedures and follow-up examinations.

**Exclusion criteria:**

1. Patients diagnosed with secondary knee osteoarthritis.

2. Patients with other diseases that impair knee function or affect the joints (such as acute joint injury caused by trauma, rheumatoid arthritis, metabolic bone disease, psoriatic arthritis, gouty arthritis, symptomatic cartilage calcification, osteonecrosis or active infection, etc.).

3. Patients who have previously used stem cell drugs for treatment.

4. Patients who have undergone major surgery, arthroplasty or arthroscopy on the study side of the knee within 24 weeks before signing the informed consent, or plan to undergo surgical treatment on the study side of the knee during the study.

5. Skin diseases or skin infections around the proposed injection site.

6. Patients with malignant tumors or a history of malignant tumors within 5 years before signing the informed consent.

7. Patients with a clear history of mental disorders, or a history of abuse of psychotropic drugs or drug abuse.

8. HBeAg-positive chronic hepatitis B patients (defined in the 2015 edition of the Guidelines for the Prevention and Treatment of Chronic Hepatitis B), hepatitis C virus infected patients, human immunodeficiency virus antibody positive, syphilis Treponema pallidum antibody positive patients.

9. Pregnant or lactating female patients, or patients (including males and females) who refuse to use contraceptive measures (abstinence, physical contraception, or hormonal contraceptives started 3 months before enrollment) during the study period and within 6 months after the end of the 5th visit.

10. Patients who participated in any other clinical trials within 3 months before signing the informed consent form.

11. Body mass index (BMI) ≥35kg/m2;

12. Patients who are judged by the investigator to be unsuitable for participation in this trial for other reasons.

**2. Table S1 : Effectiveness was compared at different times in the follow-up cycle of different dose groups**

**【By dosage】**

**low-dose group**

**Multiple comparisons**

LSD

| Dependent Variable | (I) DAY | (J) DAY | Mean Difference (I-J) | Standard error | Sig. | 95% Confidence Interval | |
| --- | --- | --- | --- | --- | --- | --- | --- |
|  |  |  |  |  |  | Lower limit | Upper limit |
| Total Score | 0 | 21 | 0.03 | 0.699 | 0.968 | -1.37 | 1.42 |
|  |  | 42 | -0.87 | 0.699 | 0.218 | -2.27 | 0.53 |
|  |  | 90 | -1.22 | 0.699 | 0.085 | -2.62 | 0.17 |
|  |  | 270 | -1.24 | 0.699 | 0.08 | -2.64 | 0.15 |
|  | 21 | 0 | -0.03 | 0.699 | 0.968 | -1.42 | 1.37 |
|  |  | 42 | -0.9 | 0.699 | 0.204 | -2.29 | 0.5 |
|  |  | 90 | -1.25 | 0.699 | 0.078 | -2.65 | 0.14 |
|  |  | 270 | -1.27 | 0.699 | 0.073 | -2.67 | 0.12 |
|  | 42 | 0 | 0.87 | 0.699 | 0.218 | -0.53 | 2.27 |
|  |  | 21 | 0.9 | 0.699 | 0.204 | -0.5 | 2.29 |
|  |  | 90 | -0.35 | 0.699 | 0.615 | -1.75 | 1.04 |
|  |  | 270 | -0.37 | 0.699 | 0.594 | -1.77 | 1.02 |
|  | 90 | 0 | 1.22 | 0.699 | 0.085 | -0.17 | 2.62 |
|  |  | 21 | 1.25 | 0.699 | 0.078 | -0.14 | 2.65 |
|  |  | 42 | 0.35 | 0.699 | 0.615 | -1.04 | 1.75 |
|  |  | 270 | -0.02 | 0.699 | 0.976 | -1.42 | 1.38 |
|  | 270 | 0 | 1.24 | 0.699 | 0.08 | -0.15 | 2.64 |
|  |  | 21 | 1.27 | 0.699 | 0.073 | -0.12 | 2.67 |
|  |  | 42 | 0.37 | 0.699 | 0.594 | -1.02 | 1.77 |
|  |  | 90 | 0.02 | 0.699 | 0.976 | -1.38 | 1.42 |
| Pain | 0 | 21 | -0.19 | 0.624 | 0.764 | -1.43 | 1.06 |
|  |  | 42 | -0.96 | 0.624 | 0.129 | -2.2 | 0.29 |
|  |  | 90 | -1.52* | 0.624 | 0.017 | -2.77 | -0.28 |
|  |  | 270 | -1.47* | 0.624 | 0.022 | -2.71 | -0.22 |
|  | 21 | 0 | 0.19 | 0.624 | 0.764 | -1.06 | 1.43 |
|  |  | 42 | -0.77 | 0.624 | 0.221 | -2.02 | 0.48 |
|  |  | 90 | -1.33* | 0.624 | 0.036 | -2.58 | -0.09 |
|  |  | 270 | -1.28* | 0.624 | 0.044 | -2.52 | -0.03 |
|  | 42 | 0 | 0.96 | 0.624 | 0.129 | -0.29 | 2.2 |
|  |  | 21 | 0.77 | 0.624 | 0.221 | -0.48 | 2.02 |
|  |  | 90 | -0.56 | 0.624 | 0.369 | -1.81 | 0.68 |
|  |  | 270 | -0.51 | 0.624 | 0.419 | -1.75 | 0.74 |
|  | 90 | 0 | 1.52* | 0.624 | 0.017 | 0.28 | 2.77 |
|  |  | 21 | 1.33* | 0.624 | 0.036 | 0.09 | 2.58 |
|  |  | 42 | 0.56 | 0.624 | 0.369 | -0.68 | 1.81 |
|  |  | 270 | 0.06 | 0.624 | 0.928 | -1.19 | 1.3 |
|  | 270 | 0 | 1.47* | 0.624 | 0.022 | 0.22 | 2.71 |
|  |  | 21 | 1.28* | 0.624 | 0.044 | 0.03 | 2.52 |
|  |  | 42 | 0.51 | 0.624 | 0.419 | -0.74 | 1.75 |
|  |  | 90 | -0.06 | 0.624 | 0.928 | -1.3 | 1.19 |
| Stiffness | 0 | 21 | -0.14 | 0.651 | 0.827 | -1.44 | 1.16 |
|  |  | 42 | -0.61 | 0.651 | 0.354 | -1.91 | 0.69 |
|  |  | 90 | -1.04 | 0.651 | 0.116 | -2.34 | 0.26 |
|  |  | 270 | -0.71 | 0.651 | 0.276 | -2.01 | 0.59 |
|  | 21 | 0 | 0.14 | 0.651 | 0.827 | -1.16 | 1.44 |
|  |  | 42 | -0.46 | 0.651 | 0.478 | -1.76 | 0.84 |
|  |  | 90 | -0.89 | 0.651 | 0.175 | -2.19 | 0.41 |
|  |  | 270 | -0.57 | 0.651 | 0.383 | -1.87 | 0.73 |
|  | 42 | 0 | 0.61 | 0.651 | 0.354 | -0.69 | 1.91 |
|  |  | 21 | 0.46 | 0.651 | 0.478 | -0.84 | 1.76 |
|  |  | 90 | -0.43 | 0.651 | 0.512 | -1.73 | 0.87 |
|  |  | 270 | -0.11 | 0.651 | 0.87 | -1.41 | 1.19 |
|  | 90 | 0 | 1.04 | 0.651 | 0.116 | -0.26 | 2.34 |
|  |  | 21 | 0.89 | 0.651 | 0.175 | -0.41 | 2.19 |
|  |  | 42 | 0.43 | 0.651 | 0.512 | -0.87 | 1.73 |
|  |  | 270 | 0.32 | 0.651 | 0.623 | -0.98 | 1.62 |
|  | 270 | 0 | 0.71 | 0.651 | 0.276 | -0.59 | 2.01 |
|  |  | 21 | 0.57 | 0.651 | 0.383 | -0.73 | 1.87 |
|  |  | 42 | 0.11 | 0.651 | 0.87 | -1.19 | 1.41 |
|  |  | 90 | -0.32 | 0.651 | 0.623 | -1.62 | 0.98 |
| Difficulty in Daily Life | 0 | 21 | -0.1 | 0.762 | 0.892 | -1.63 | 1.42 |
|  |  | 42 | 0.87 | 0.762 | 0.26 | -0.66 | 2.39 |
|  |  | 90 | 1.15 | 0.762 | 0.136 | -0.37 | 2.67 |
|  |  | 270 | 1.23 | 0.762 | 0.11 | -0.29 | 2.76 |
|  | 21 | 0 | 0.1 | 0.762 | 0.892 | -1.42 | 1.63 |
|  |  | 42 | 0.97 | 0.762 | 0.208 | -0.55 | 2.49 |
|  |  | 90 | 1.25 | 0.762 | 0.105 | -0.27 | 2.78 |
|  |  | 270 | 1.34 | 0.762 | 0.084 | -0.18 | 2.86 |
|  | 42 | 0 | -0.87 | 0.762 | 0.26 | -2.39 | 0.66 |
|  |  | 21 | -0.97 | 0.762 | 0.208 | -2.49 | 0.55 |
|  |  | 90 | 0.28 | 0.762 | 0.711 | -1.24 | 1.81 |
|  |  | 270 | 0.37 | 0.762 | 0.63 | -1.15 | 1.89 |
|  | 90 | 0 | -1.15 | 0.762 | 0.136 | -2.67 | 0.37 |
|  |  | 21 | -1.25 | 0.762 | 0.105 | -2.78 | 0.27 |
|  |  | 42 | -0.28 | 0.762 | 0.711 | -1.81 | 1.24 |
|  |  | 270 | 0.09 | 0.762 | 0.911 | -1.44 | 1.61 |
|  | 270 | 0 | -1.23 | 0.762 | 0.11 | -2.76 | 0.29 |
|  |  | 21 | -1.34 | 0.762 | 0.084 | -2.86 | 0.18 |
|  |  | 42 | -0.37 | 0.762 | 0.63 | -1.89 | 1.15 |
|  |  | 90 | -0.09 | 0.762 | 0.911 | -1.61 | 1.44 |

Based on observed means.

The error term is MeanSquare(Error) = 4.067.

*. The mean difference is significant at the .05 level.

**mid-dose group**

**Multiple comparisons**

LSD

| Dependent Variable | (I) DAY | (J) DAY | Mean Difference (I-J) | Standard error | Sig. | 95% Confidence Interval | |
| --- | --- | --- | --- | --- | --- | --- | --- |
|  |  |  |  |  |  | Lower limit | Upper limit |
| Total Score | 0 | 21 | -0.1 | 0.789 | 0.902 | -1.67 | 1.48 |
|  |  | 42 | -1.13 | 0.789 | 0.157 | -2.71 | 0.45 |
|  |  | 90 | -1.42 | 0.835 | 0.094 | -3.09 | 0.25 |
|  |  | 270 | -1.34 | 0.855 | 0.123 | -3.05 | 0.37 |
|  | 21 | 0 | 0.1 | 0.789 | 0.902 | -1.48 | 1.67 |
|  |  | 42 | -1.03 | 0.775 | 0.188 | -2.58 | 0.52 |
|  |  | 90 | -1.32 | 0.822 | 0.113 | -2.96 | 0.32 |
|  |  | 270 | -1.24 | 0.843 | 0.146 | -2.93 | 0.44 |
|  | 42 | 0 | 1.13 | 0.789 | 0.157 | -0.45 | 2.71 |
|  |  | 21 | 1.03 | 0.775 | 0.188 | -0.52 | 2.58 |
|  |  | 90 | -0.29 | 0.822 | 0.727 | -1.93 | 1.36 |
|  |  | 270 | -0.21 | 0.843 | 0.805 | -1.89 | 1.48 |
|  | 90 | 0 | 1.42 | 0.835 | 0.094 | -0.25 | 3.09 |
|  |  | 21 | 1.32 | 0.822 | 0.113 | -0.32 | 2.96 |
|  |  | 42 | 0.29 | 0.822 | 0.727 | -1.36 | 1.93 |
|  |  | 270 | 0.08 | 0.886 | 0.929 | -1.69 | 1.85 |
|  | 270 | 0 | 1.34 | 0.855 | 0.123 | -0.37 | 3.05 |
|  |  | 21 | 1.24 | 0.843 | 0.146 | -0.44 | 2.93 |
|  |  | 42 | 0.21 | 0.843 | 0.805 | -1.48 | 1.89 |
|  |  | 90 | -0.08 | 0.886 | 0.929 | -1.85 | 1.69 |
| Pain | 0 | 21 | -0.25 | 0.812 | 0.757 | -1.88 | 1.37 |
|  |  | 42 | -0.9 | 0.812 | 0.272 | -2.52 | 0.72 |
|  |  | 90 | -1.52 | 0.86 | 0.082 | -3.24 | 0.2 |
|  |  | 270 | -1.39 | 0.88 | 0.121 | -3.15 | 0.37 |
|  | 21 | 0 | 0.25 | 0.812 | 0.757 | -1.37 | 1.88 |
|  |  | 42 | -0.65 | 0.798 | 0.419 | -2.24 | 0.95 |
|  |  | 90 | -1.27 | 0.846 | 0.139 | -2.96 | 0.42 |
|  |  | 270 | -1.13 | 0.867 | 0.196 | -2.87 | 0.6 |
|  | 42 | 0 | 0.9 | 0.812 | 0.272 | -0.72 | 2.52 |
|  |  | 21 | 0.65 | 0.798 | 0.419 | -0.95 | 2.24 |
|  |  | 90 | -0.62 | 0.846 | 0.468 | -2.31 | 1.07 |
|  |  | 270 | -0.48 | 0.867 | 0.578 | -2.22 | 1.25 |
|  | 90 | 0 | 1.52 | 0.86 | 0.082 | -0.2 | 3.24 |
|  |  | 21 | 1.27 | 0.846 | 0.139 | -0.42 | 2.96 |
|  |  | 42 | 0.62 | 0.846 | 0.468 | -1.07 | 2.31 |
|  |  | 270 | 0.13 | 0.912 | 0.884 | -1.69 | 1.96 |
|  | 270 | 0 | 1.39 | 0.88 | 0.121 | -0.37 | 3.15 |
|  |  | 21 | 1.13 | 0.867 | 0.196 | -0.6 | 2.87 |
|  |  | 42 | 0.48 | 0.867 | 0.578 | -1.25 | 2.22 |
|  |  | 90 | -0.13 | 0.912 | 0.884 | -1.96 | 1.69 |
| Stiffness | 0 | 21 | 0.69 | 0.941 | 0.466 | -1.19 | 2.57 |
|  |  | 42 | -0.68 | 0.941 | 0.475 | -2.56 | 1.21 |
|  |  | 90 | -0.73 | 0.997 | 0.469 | -2.72 | 1.27 |
|  |  | 270 | -0.46 | 1.021 | 0.653 | -2.5 | 1.58 |
|  | 21 | 0 | -0.69 | 0.941 | 0.466 | -2.57 | 1.19 |
|  |  | 42 | -1.37 | 0.925 | 0.145 | -3.22 | 0.48 |
|  |  | 90 | -1.42 | 0.981 | 0.154 | -3.38 | 0.54 |
|  |  | 270 | -1.15 | 1.006 | 0.257 | -3.16 | 0.86 |
|  | 42 | 0 | 0.68 | 0.941 | 0.475 | -1.21 | 2.56 |
|  |  | 21 | 1.37 | 0.925 | 0.145 | -0.48 | 3.22 |
|  |  | 90 | -0.05 | 0.981 | 0.96 | -2.01 | 1.91 |
|  |  | 270 | 0.22 | 1.006 | 0.831 | -1.79 | 2.23 |
|  | 90 | 0 | 0.73 | 0.997 | 0.469 | -1.27 | 2.72 |
|  |  | 21 | 1.42 | 0.981 | 0.154 | -0.54 | 3.38 |
|  |  | 42 | 0.05 | 0.981 | 0.96 | -1.91 | 2.01 |
|  |  | 270 | 0.27 | 1.057 | 0.803 | -1.85 | 2.38 |
|  | 270 | 0 | 0.46 | 1.021 | 0.653 | -1.58 | 2.5 |
|  |  | 21 | 1.15 | 1.006 | 0.257 | -0.86 | 3.16 |
|  |  | 42 | -0.22 | 1.006 | 0.831 | -2.23 | 1.79 |
|  |  | 90 | -0.27 | 1.057 | 0.803 | -2.38 | 1.85 |
| Difficulty in Daily Life | 0 | 21 | 0.42 | 0.806 | 0.603 | -1.19 | 2.03 |
|  |  | 42 | 1.49 | 0.806 | 0.07 | -0.13 | 3.1 |
|  |  | 90 | 1.72^*^ | 0.854 | 0.049 | 0.01 | 3.42 |
|  |  | 270 | 1.67 | 0.874 | 0.061 | -0.08 | 3.42 |
|  | 21 | 0 | -0.42 | 0.806 | 0.603 | -2.03 | 1.19 |
|  |  | 42 | 1.06 | 0.792 | 0.184 | -0.52 | 2.65 |
|  |  | 90 | 1.29 | 0.84 | 0.128 | -0.39 | 2.97 |
|  |  | 270 | 1.25 | 0.861 | 0.152 | -0.47 | 2.97 |
|  | 42 | 0 | -1.49 | 0.806 | 0.07 | -3.1 | 0.13 |
|  |  | 21 | -1.06 | 0.792 | 0.184 | -2.65 | 0.52 |
|  |  | 90 | 0.23 | 0.84 | 0.785 | -1.45 | 1.91 |
|  |  | 270 | 0.19 | 0.861 | 0.831 | -1.54 | 1.91 |
|  | 90 | 0 | -1.72^*^ | 0.854 | 0.049 | -3.42 | -0.01 |
|  |  | 21 | -1.29 | 0.84 | 0.128 | -2.97 | 0.39 |
|  |  | 42 | -0.23 | 0.84 | 0.785 | -1.91 | 1.45 |
|  |  | 270 | -0.05 | 0.906 | 0.96 | -1.86 | 1.76 |
|  | 270 | 0 | -1.67 | 0.874 | 0.061 | -3.42 | 0.08 |
|  |  | 21 | -1.25 | 0.861 | 0.152 | -2.97 | 0.47 |
|  |  | 42 | -0.19 | 0.861 | 0.831 | -1.91 | 1.54 |
|  |  | 90 | 0.05 | 0.906 | 0.96 | -1.76 | 1.86 |

Based on the observed mean.

The error term is MeanSquare(Error) = 4.708.

*. The mean difference is significant at the .05 level.

**high-dose group**

**Multiple comparisons**

LSD

| Dependent Variable | (I) DAY | (J) DAY | Mean Difference (I-J) | Standard error | Sig. | 95% Confidence Interval | |
| --- | --- | --- | --- | --- | --- | --- | --- |
|  |  |  |  |  |  | Lower limit | Upper limit |
| Total Score | 0 | 21 | -0.1 | 0.789 | 0.902 | -1.67 | 1.48 |
|  |  | 42 | -1.13 | 0.789 | 0.157 | -2.71 | 0.45 |
|  |  | 90 | -1.42 | 0.835 | 0.094 | -3.09 | 0.25 |
|  |  | 270 | -1.34 | 0.855 | 0.123 | -3.05 | 0.37 |
|  | 21 | 0 | 0.1 | 0.789 | 0.902 | -1.48 | 1.67 |
|  |  | 42 | -1.03 | 0.775 | 0.188 | -2.58 | 0.52 |
|  |  | 90 | -1.32 | 0.822 | 0.113 | -2.96 | 0.32 |
|  |  | 270 | -1.24 | 0.843 | 0.146 | -2.93 | 0.44 |
|  | 42 | 0 | 1.13 | 0.789 | 0.157 | -0.45 | 2.71 |
|  |  | 21 | 1.03 | 0.775 | 0.188 | -0.52 | 2.58 |
|  |  | 90 | -0.29 | 0.822 | 0.727 | -1.93 | 1.36 |
|  |  | 270 | -0.21 | 0.843 | 0.805 | -1.89 | 1.48 |
|  | 90 | 0 | 1.42 | 0.835 | 0.094 | -0.25 | 3.09 |
|  |  | 21 | 1.32 | 0.822 | 0.113 | -0.32 | 2.96 |
|  |  | 42 | 0.29 | 0.822 | 0.727 | -1.36 | 1.93 |
|  |  | 270 | 0.08 | 0.886 | 0.929 | -1.69 | 1.85 |
|  | 270 | 0 | 1.34 | 0.855 | 0.123 | -0.37 | 3.05 |
|  |  | 21 | 1.24 | 0.843 | 0.146 | -0.44 | 2.93 |
|  |  | 42 | 0.21 | 0.843 | 0.805 | -1.48 | 1.89 |
|  |  | 90 | -0.08 | 0.886 | 0.929 | -1.85 | 1.69 |
| Pain | 0 | 21 | -0.25 | 0.812 | 0.757 | -1.88 | 1.37 |
|  |  | 42 | -0.9 | 0.812 | 0.272 | -2.52 | 0.72 |
|  |  | 90 | -1.52 | 0.86 | 0.082 | -3.24 | 0.2 |
|  |  | 270 | -1.39 | 0.88 | 0.121 | -3.15 | 0.37 |
|  | 21 | 0 | 0.25 | 0.812 | 0.757 | -1.37 | 1.88 |
|  |  | 42 | -0.65 | 0.798 | 0.419 | -2.24 | 0.95 |
|  |  | 90 | -1.27 | 0.846 | 0.139 | -2.96 | 0.42 |
|  |  | 270 | -1.13 | 0.867 | 0.196 | -2.87 | 0.6 |
|  | 42 | 0 | 0.9 | 0.812 | 0.272 | -0.72 | 2.52 |
|  |  | 21 | 0.65 | 0.798 | 0.419 | -0.95 | 2.24 |
|  |  | 90 | -0.62 | 0.846 | 0.468 | -2.31 | 1.07 |
|  |  | 270 | -0.48 | 0.867 | 0.578 | -2.22 | 1.25 |
|  | 90 | 0 | 1.52 | 0.86 | 0.082 | -0.2 | 3.24 |
|  |  | 21 | 1.27 | 0.846 | 0.139 | -0.42 | 2.96 |
|  |  | 42 | 0.62 | 0.846 | 0.468 | -1.07 | 2.31 |
|  |  | 270 | 0.13 | 0.912 | 0.884 | -1.69 | 1.96 |
|  | 270 | 0 | 1.39 | 0.88 | 0.121 | -0.37 | 3.15 |
|  |  | 21 | 1.13 | 0.867 | 0.196 | -0.6 | 2.87 |
|  |  | 42 | 0.48 | 0.867 | 0.578 | -1.25 | 2.22 |
|  |  | 90 | -0.13 | 0.912 | 0.884 | -1.96 | 1.69 |
| Stiffness | 0 | 21 | 0.69 | 0.941 | 0.466 | -1.19 | 2.57 |
|  |  | 42 | -0.68 | 0.941 | 0.475 | -2.56 | 1.21 |
|  |  | 90 | -0.73 | 0.997 | 0.469 | -2.72 | 1.27 |
|  |  | 270 | -0.46 | 1.021 | 0.653 | -2.5 | 1.58 |
|  | 21 | 0 | -0.69 | 0.941 | 0.466 | -2.57 | 1.19 |
|  |  | 42 | -1.37 | 0.925 | 0.145 | -3.22 | 0.48 |
|  |  | 90 | -1.42 | 0.981 | 0.154 | -3.38 | 0.54 |
|  |  | 270 | -1.15 | 1.006 | 0.257 | -3.16 | 0.86 |
|  | 42 | 0 | 0.68 | 0.941 | 0.475 | -1.21 | 2.56 |
|  |  | 21 | 1.37 | 0.925 | 0.145 | -0.48 | 3.22 |
|  |  | 90 | -0.05 | 0.981 | 0.96 | -2.01 | 1.91 |
|  |  | 270 | 0.22 | 1.006 | 0.831 | -1.79 | 2.23 |
|  | 90 | 0 | 0.73 | 0.997 | 0.469 | -1.27 | 2.72 |
|  |  | 21 | 1.42 | 0.981 | 0.154 | -0.54 | 3.38 |
|  |  | 42 | 0.05 | 0.981 | 0.96 | -1.91 | 2.01 |
|  |  | 270 | 0.27 | 1.057 | 0.803 | -1.85 | 2.38 |
|  | 270 | 0 | 0.46 | 1.021 | 0.653 | -1.58 | 2.5 |
|  |  | 21 | 1.15 | 1.006 | 0.257 | -0.86 | 3.16 |
|  |  | 42 | -0.22 | 1.006 | 0.831 | -2.23 | 1.79 |
|  |  | 90 | -0.27 | 1.057 | 0.803 | -2.38 | 1.85 |
| Difficulty in Daily Life | 0 | 21 | 0.42 | 0.806 | 0.603 | -1.19 | 2.03 |
|  |  | 42 | 1.49 | 0.806 | 0.07 | -0.13 | 3.1 |
|  |  | 90 | 1.72^*^ | 0.854 | 0.049 | 0.01 | 3.42 |
|  |  | 270 | 1.67 | 0.874 | 0.061 | -0.08 | 3.42 |
|  | 21 | 0 | -0.42 | 0.806 | 0.603 | -2.03 | 1.19 |
|  |  | 42 | 1.06 | 0.792 | 0.184 | -0.52 | 2.65 |
|  |  | 90 | 1.29 | 0.84 | 0.128 | -0.39 | 2.97 |
|  |  | 270 | 1.25 | 0.861 | 0.152 | -0.47 | 2.97 |
|  | 42 | 0 | -1.49 | 0.806 | 0.07 | -3.1 | 0.13 |
|  |  | 21 | -1.06 | 0.792 | 0.184 | -2.65 | 0.52 |
|  |  | 90 | 0.23 | 0.84 | 0.785 | -1.45 | 1.91 |
|  |  | 270 | 0.19 | 0.861 | 0.831 | -1.54 | 1.91 |
|  | 90 | 0 | -1.72^*^ | 0.854 | 0.049 | -3.42 | -0.01 |
|  |  | 21 | -1.29 | 0.84 | 0.128 | -2.97 | 0.39 |
|  |  | 42 | -0.23 | 0.84 | 0.785 | -1.91 | 1.45 |
|  |  | 270 | -0.05 | 0.906 | 0.96 | -1.86 | 1.76 |
|  | 270 | 0 | -1.67 | 0.874 | 0.061 | -3.42 | 0.08 |
|  |  | 21 | -1.25 | 0.861 | 0.152 | -2.97 | 0.47 |
|  |  | 42 | -0.19 | 0.861 | 0.831 | -1.91 | 1.54 |
|  |  | 90 | 0.05 | 0.906 | 0.96 | -1.76 | 1.86 |

Based on observed means.

The error term is MeanSquare(Error) = 1.965.

*. The mean difference is significant at the .05 level.

**3. Table S2 : Effectiveness was compared in each dose group at different follow-up times.**

【By time】

**Day 0**

LSD

| Dependent Variable | (I) Dose | (J) Dose | Mean Difference (I-J) | Standard error | Sig. | 95% Confidence Interval | |
| --- | --- | --- | --- | --- | --- | --- | --- |
|  |  |  |  |  |  | Lower limit | Upper limit |
| Total Score | 3 | 4 | 0.73 | 0.723 | 0.32 | -0.73 | 2.19 |
|  |  | 5 | 0.88 | 0.711 | 0.225 | -0.56 | 2.31 |
|  | 4 | 3 | -0.73 | 0.723 | 0.32 | -2.19 | 0.73 |
|  |  | 5 | 0.15 | 0.711 | 0.836 | -1.29 | 1.58 |
|  | 5 | 3 | -0.88 | 0.711 | 0.225 | -2.31 | 0.56 |
|  |  | 4 | -0.15 | 0.711 | 0.836 | -1.58 | 1.29 |
| Pain | 3 | 4 | 1 | 0.828 | 0.236 | -0.68 | 2.67 |
|  |  | 5 | 2.34^*^ | 0.814 | 0.006 | 0.7 | 3.99 |
|  | 4 | 3 | -1 | 0.828 | 0.236 | -2.67 | 0.68 |
|  |  | 5 | 1.34 | 0.814 | 0.106 | -0.3 | 2.99 |
|  | 5 | 3 | -2.34^*^ | 0.814 | 0.006 | -3.99 | -0.7 |
|  |  | 4 | -1.34 | 0.814 | 0.106 | -2.99 | 0.3 |
| Stiffness | 3 | 4 | 0.93 | 0.916 | 0.317 | -0.92 | 2.78 |
|  |  | 5 | 1.25 | 0.9 | 0.172 | -0.57 | 3.07 |
|  | 4 | 3 | -0.93 | 0.916 | 0.317 | -2.78 | 0.92 |
|  |  | 5 | 0.32 | 0.9 | 0.721 | -1.5 | 2.14 |
|  | 5 | 3 | -1.25 | 0.9 | 0.172 | -3.07 | 0.57 |
|  |  | 4 | -0.32 | 0.9 | 0.721 | -2.14 | 1.5 |
| Difficulty in Daily Life | 3 | 4 | -0.88 | 0.738 | 0.24 | -2.37 | 0.61 |
|  |  | 5 | -0.44 | 0.725 | 0.547 | -1.91 | 1.03 |
|  | 4 | 3 | 0.88 | 0.738 | 0.24 | -0.61 | 2.37 |
|  |  | 5 | 0.44 | 0.725 | 0.548 | -1.03 | 1.91 |
|  | 5 | 3 | 0.44 | 0.725 | 0.547 | -1.03 | 1.91 |
|  |  | 4 | -0.44 | 0.725 | 0.548 | -1.91 | 1.03 |

Based on observed means.

The error term is MeanSquare(Error) = 3.810.

*. The mean difference is significant at the .05 level.

**Day 21**

**Multiple comparisons**

LSD

| Dependent Variable | (I) Dose | (J) Dose | Mean Difference (I-J) | Standard error | Sig. | 95% Confidence Interval | |
| --- | --- | --- | --- | --- | --- | --- | --- |
|  |  |  |  |  |  | Lower limit | Upper limit |
| Total Score | 3 | 4 | 0.6 | 0.758 | 0.431 | -0.93 | 2.13 |
|  |  | 5 | -0.33 | 0.758 | 0.669 | -1.86 | 1.21 |
|  | 4 | 3 | -0.6 | 0.758 | 0.431 | -2.13 | 0.93 |
|  |  | 5 | -0.93 | 0.745 | 0.22 | -2.43 | 0.58 |
|  | 5 | 3 | 0.33 | 0.758 | 0.669 | -1.21 | 1.86 |
|  |  | 4 | 0.93 | 0.745 | 0.22 | -0.58 | 2.43 |
| Pain | 3 | 4 | 0.93 | 0.797 | 0.249 | -0.68 | 2.54 |
|  |  | 5 | 0.44 | 0.797 | 0.583 | -1.17 | 2.05 |
|  | 4 | 3 | -0.93 | 0.797 | 0.249 | -2.54 | 0.68 |
|  |  | 5 | -0.49 | 0.783 | 0.534 | -2.07 | 1.09 |
|  | 5 | 3 | -0.44 | 0.797 | 0.583 | -2.05 | 1.17 |
|  |  | 4 | 0.49 | 0.783 | 0.534 | -1.09 | 2.07 |
| Stiffness | 3 | 4 | 1.76 | 0.906 | 0.059 | -0.07 | 3.59 |
|  |  | 5 | 0.43 | 0.906 | 0.639 | -1.4 | 2.26 |
|  | 4 | 3 | -1.76 | 0.906 | 0.059 | -3.59 | 0.07 |
|  |  | 5 | -1.33 | 0.89 | 0.142 | -3.13 | 0.46 |
|  | 5 | 3 | -0.43 | 0.906 | 0.639 | -2.26 | 1.4 |
|  |  | 4 | 1.33 | 0.89 | 0.142 | -0.46 | 3.13 |
| Difficulty in Daily Life | 3 | 4 | -0.35 | 0.773 | 0.649 | -1.91 | 1.21 |
|  |  | 5 | 0.61 | 0.773 | 0.432 | -0.95 | 2.17 |
|  | 4 | 3 | 0.35 | 0.773 | 0.649 | -1.21 | 1.91 |
|  |  | 5 | 0.97 | 0.759 | 0.21 | -0.57 | 2.5 |
|  | 5 | 3 | -0.61 | 0.773 | 0.432 | -2.17 | 0.95 |
|  |  | 4 | -0.97 | 0.759 | 0.21 | -2.5 | 0.57 |

Based on observed means.

The error term is MeanSquare(Error) = 4.322.

**Day 42**

**Multiple comparisons**

LSD

| Dependent Variable | (I) Dose | (J) Dose | Mean Difference (I-J) | Standard error | Sig. | 95% Confidence Interval | |
| --- | --- | --- | --- | --- | --- | --- | --- |
|  |  |  |  |  |  | Lower limit | Upper limit |
| Total Score | 3 | 4 | 0.47 | 0.618 | 0.453 | -0.78 | 1.72 |
|  |  | 5 | -0.23 | 0.618 | 0.707 | -1.48 | 1.01 |
|  | 4 | 3 | -0.47 | 0.618 | 0.453 | -1.72 | 0.78 |
|  |  | 5 | -0.7 | 0.607 | 0.254 | -1.93 | 0.52 |
|  | 5 | 3 | 0.23 | 0.618 | 0.707 | -1.01 | 1.48 |
|  |  | 4 | 0.7 | 0.607 | 0.254 | -0.52 | 1.93 |
| Pain | 3 | 4 | 1.05 | 0.708 | 0.144 | -0.38 | 2.48 |
|  |  | 5 | 0.25 | 0.708 | 0.729 | -1.18 | 1.68 |
|  | 4 | 3 | -1.05 | 0.708 | 0.144 | -2.48 | 0.38 |
|  |  | 5 | -0.81 | 0.695 | 0.253 | -2.21 | 0.6 |
|  | 5 | 3 | -0.25 | 0.708 | 0.729 | -1.68 | 1.18 |
|  |  | 4 | 0.81 | 0.695 | 0.253 | -0.6 | 2.21 |
| Stiffness | 3 | 4 | 0.86 | 0.599 | 0.159 | -0.35 | 2.07 |
|  |  | 5 | 0.36 | 0.599 | 0.552 | -0.85 | 1.57 |
|  | 4 | 3 | -0.86 | 0.599 | 0.159 | -2.07 | 0.35 |
|  |  | 5 | -0.5 | 0.589 | 0.401 | -1.69 | 0.69 |
|  | 5 | 3 | -0.36 | 0.599 | 0.552 | -1.57 | 0.85 |
|  |  | 4 | 0.5 | 0.589 | 0.401 | -0.69 | 1.69 |
| Difficulty in Daily Life | 3 | 4 | -0.26 | 0.616 | 0.676 | -1.5 | 0.99 |
|  |  | 5 | 0.43 | 0.616 | 0.491 | -0.82 | 1.67 |
|  | 4 | 3 | 0.26 | 0.616 | 0.676 | -0.99 | 1.5 |
|  |  | 5 | 0.69 | 0.606 | 0.262 | -0.53 | 1.91 |
|  | 5 | 3 | -0.43 | 0.616 | 0.491 | -1.67 | 0.82 |
|  |  | 4 | -0.69 | 0.606 | 0.262 | -1.91 | 0.53 |

Based on observed means.

The error term is MeanSquare(Error) = 2.752.

**Day 90**

**Multiple comparisons**

LSD

| Dependent Variable | (I) Dose | (J) Dose | Mean Difference (I-J) | Standard error | Sig. | 95% Confidence Interval | |
| --- | --- | --- | --- | --- | --- | --- | --- |
|  |  |  |  |  |  | Lower limit | Upper limit |
| Total Score | 3 | 4 | 0.53 | 0.673 | 0.433 | -0.83 | 1.9 |
|  |  | 5 | -0.33 | 0.647 | 0.614 | -1.64 | 0.98 |
|  | 4 | 3 | -0.53 | 0.673 | 0.433 | -1.9 | 0.83 |
|  |  | 5 | -0.86 | 0.673 | 0.208 | -2.23 | 0.5 |
|  | 5 | 3 | 0.33 | 0.647 | 0.614 | -0.98 | 1.64 |
|  |  | 4 | 0.86 | 0.673 | 0.208 | -0.5 | 2.23 |
| Pain | 3 | 4 | 1 | 0.619 | 0.115 | -0.25 | 2.25 |
|  |  | 5 | 0.38 | 0.594 | 0.531 | -0.83 | 1.58 |
|  | 4 | 3 | -1 | 0.619 | 0.115 | -2.25 | 0.25 |
|  |  | 5 | -0.62 | 0.619 | 0.32 | -1.88 | 0.63 |
|  | 5 | 3 | -0.38 | 0.594 | 0.531 | -1.58 | 0.83 |
|  |  | 4 | 0.62 | 0.619 | 0.32 | -0.63 | 1.88 |
| Stiffness | 3 | 4 | 1.24 | 0.692 | 0.082 | -0.16 | 2.64 |
|  |  | 5 | 0.57 | 0.665 | 0.396 | -0.78 | 1.92 |
|  | 4 | 3 | -1.24 | 0.692 | 0.082 | -2.64 | 0.16 |
|  |  | 5 | -0.67 | 0.692 | 0.342 | -2.07 | 0.74 |
|  | 5 | 3 | -0.57 | 0.665 | 0.396 | -1.92 | 0.78 |
|  |  | 4 | 0.67 | 0.692 | 0.342 | -0.74 | 2.07 |
| Difficulty in Daily Life | 3 | 4 | -0.31 | 0.734 | 0.672 | -1.8 | 1.17 |
|  |  | 5 | 0.62 | 0.705 | 0.385 | -0.81 | 2.05 |
|  | 4 | 3 | 0.31 | 0.734 | 0.672 | -1.17 | 1.8 |
|  |  | 5 | 0.93 | 0.734 | 0.212 | -0.55 | 2.42 |
|  | 5 | 3 | -0.62 | 0.705 | 0.385 | -2.05 | 0.81 |
|  |  | 4 | -0.93 | 0.734 | 0.212 | -2.42 | 0.55 |

Based on observed means.

The error term is MeanSquare(Error) = 3.479.

**Day 270**

**Multiple comparisons**

LSD

| Dependent Variable | (I) Dose | (J) Dose | Mean Difference (I-J) | Standard error | Sig. | 95% Confidence Interval | |
| --- | --- | --- | --- | --- | --- | --- | --- |
|  |  |  |  |  |  | Lower limit | Upper limit |
| Total Score | 3 | 4 | 0.63 | 0.694 | 0.367 | -0.77 | 2.04 |
|  |  | 5 | -0.4 | 0.651 | 0.547 | -1.71 | 0.92 |
|  | 4 | 3 | -0.63 | 0.694 | 0.367 | -2.04 | 0.77 |
|  |  | 5 | -1.03 | 0.694 | 0.146 | -2.44 | 0.38 |
|  | 5 | 3 | 0.4 | 0.651 | 0.547 | -0.92 | 1.71 |
|  |  | 4 | 1.03 | 0.694 | 0.146 | -0.38 | 2.44 |
| Pain | 3 | 4 | 1.08 | 0.652 | 0.108 | -0.25 | 2.4 |
|  |  | 5 | 0.11 | 0.612 | 0.855 | -1.13 | 1.35 |
|  | 4 | 3 | -1.08 | 0.652 | 0.108 | -2.4 | 0.25 |
|  |  | 5 | -0.96 | 0.652 | 0.148 | -2.29 | 0.36 |
|  | 5 | 3 | -0.11 | 0.612 | 0.855 | -1.35 | 1.13 |
|  |  | 4 | 0.96 | 0.652 | 0.148 | -0.36 | 2.29 |
| Stiffness | 3 | 4 | 1.18 | 0.741 | 0.12 | -0.32 | 2.69 |
|  |  | 5 | 0.14 | 0.695 | 0.838 | -1.27 | 1.55 |
|  | 4 | 3 | -1.18 | 0.741 | 0.12 | -2.69 | 0.32 |
|  |  | 5 | -1.04 | 0.741 | 0.17 | -2.54 | 0.46 |
|  | 5 | 3 | -0.14 | 0.695 | 0.838 | -1.55 | 1.27 |
|  |  | 4 | 1.04 | 0.741 | 0.17 | -0.46 | 2.54 |
| Difficulty in Daily Life | 3 | 4 | -0.44 | 0.732 | 0.548 | -1.93 | 1.04 |
|  |  | 5 | 0.59 | 0.686 | 0.395 | -0.8 | 1.98 |
|  | 4 | 3 | 0.44 | 0.732 | 0.548 | -1.04 | 1.93 |
|  |  | 5 | 1.04 | 0.732 | 0.166 | -0.45 | 2.52 |
|  | 5 | 3 | -0.59 | 0.686 | 0.395 | -1.98 | 0.8 |
|  |  | 4 | -1.04 | 0.732 | 0.166 | -2.52 | 0.45 |

Based on observed means.

The error term is MeanSquare(Error) = 3.297.
